# The Genome of the Acid Soil-Adapted Strain *Rhizobium favelukesii* OR191 Encodes Determinants for Effective Symbiotic Interaction With Both an Inverted Repeat Lacking Clade and a Phaseoloid Legume Host

**DOI:** 10.3389/fmicb.2022.735911

**Published:** 2022-04-13

**Authors:** Bertrand Eardly, Wan Adnawani Meor Osman, Julie Ardley, Jaco Zandberg, Margaret Gollagher, Peter van Berkum, Patrick Elia, Dora Marinova, Rekha Seshadri, T. B. K. Reddy, Natalia Ivanova, Amrita Pati, Tanja Woyke, Nikos Kyrpides, Matthys Loedolff, Damian W. Laird, Wayne Reeve

**Affiliations:** ^1^Berks College, Penn State University, Reading, PA, United States; ^2^Centre for Crop and Food Innovation, College of Science, Health, Engineering and Education, Food Futures Institute, Murdoch University, Murdoch, WA, Australia; ^3^Murdoch University Associate, Murdoch, WA, Australia; ^4^Sustainability and Biosecurity, Department of Primary Industries and Regional Development, South Perth, WA, Australia; ^5^Soybean Genomics and Improvement Laboratory, United States Department of Agriculture, Beltsville, MD, United States; ^6^Curtin University Sustainability Policy Institute, Curtin University, Bentley, WA, Australia; ^7^Department of Energy (DOE) Joint Genome Institute, Berkeley, CA, United States; ^8^Centre for Water Energy and Waste, Harry Butler Institute, Murdoch University, Murdoch, WA, Australia

**Keywords:** symbiotic nitrogen fixation (SNF), host—bacteria interaction, acid soils, *Medicago sativa*, *Phaseolus vulgaris*

## Abstract

Although *Medicago sativa* forms highly effective symbioses with the comparatively acid-sensitive genus *Ensifer*, its introduction into acid soils appears to have selected for symbiotic interactions with acid-tolerant *R. favelukesii* strains. *Rhizobium favelukesii* has the unusual ability of being able to nodulate and fix nitrogen, albeit sub-optimally, not only with *M. sativa* but also with the promiscuous host *Phaseolus vulgaris*. Here we describe the genome of *R. favelukesii* OR191 and genomic features important for the symbiotic interaction with both of these hosts. The OR191 draft genome contained acid adaptation loci, including the highly acid-inducible *lpiA*/*acvB* operon and *olsC*, required for production of lysine- and ornithine-containing membrane lipids, respectively. The *olsC* gene was also present in other acid-tolerant *Rhizobium* strains but absent from the more acid-sensitive *Ensifer* microsymbionts. The OR191 symbiotic genes were in general more closely related to those found in *Medicago* microsymbionts. OR191 contained the *nodA*, *nodEF, nodHPQ*, and *nodL* genes for synthesis of polyunsaturated, sulfated and acetylated Nod factors that are important for symbiosis with *Medicago*, but contained a truncated *nodG*, which may decrease nodulation efficiency with *M. sativa*. OR191 contained an *E. meliloti* type BacA, which has been shown to specifically protect *Ensifer* microsymbionts from *Medicago* nodule-specific cysteine-rich peptides. The nitrogen fixation genes *nifQWZS* were present in OR191 and *P. vulgaris* microsymbionts but absent from *E. meliloti-Medicago* microsymbionts. The ability of OR191 to nodulate and fix nitrogen symbiotically with *P. vulgaris* indicates that this host has less stringent requirements for nodulation than *M. sativa* but may need rhizobial strains that possess *nifQWZS* for N_2_-fixation to occur. OR191 possessed the *exo* genes required for the biosynthesis of succinoglycan, which is required for the *Ensifer-Medicago* symbiosis. However, ^1^H-NMR spectra revealed that, in the conditions tested, OR191 exopolysaccharide did not contain a succinyl substituent but instead contained a 3-hydroxybutyrate moiety, which may affect its symbiotic performance with *Medicago* hosts. These findings provide a foundation for the genetic basis of nodulation requirements and symbiotic effectiveness with different hosts.

## Introduction

The symbiosis between legumes and root nodule bacteria (collectively known as rhizobia) is of global importance in both natural and agricultural ecosystems. It is established following a molecular dialogue between the legume and its microsymbiont that leads to infection, nodule organogenesis and the eventual endocytosis-like release of the bacteria into membrane-bound compartments in the nodule cells of the root cortex. Following this, the rhizobia differentiate into N_2_-fixing bacteroids and supply essential fixed N to the plant host.

Most current knowledge on the evolution of N_2_-fixing rhizobia is based on the comparative study of a relatively small number of symbiotically effective strains. Although this bias is understandable from an agronomic perspective, relatively little attention has been devoted to the study of strains that are poorly effective for N_2_-fixation, which may constitute the majority of rhizobial populations in soils (Bottomley and Jenkins, 1983; Segovia et al., 1991; Sullivan et al., 1996). These poorly effective strains, which are often well adapted to the soil environment, are a constraint to maximizing the productivity of the legume-rhizobia symbiosis in sustainable agricultural systems (Howieson et al., 2008). Sequencing the genomes of such strains could not only provide greater knowledge of their evolution, biogeography and symbiotic relationships, but could also contribute to an understanding of the molecular mechanisms that govern N_2_-fixation effectiveness.

The *Rhizobium favelukesii* strain OR191 was first isolated from a nodule of the perennial pasture legume alfalfa (*Medicago sativa* L.) growing in moderately acid (pH 5.5–5.7) soil at Corvallis, Oregon, in 1982, and was shown to be poorly effective on this host (Eardly et al., 1985). OR191, along with similar isolates from the same site, possessed a unique symbiotic host range; specifically, they were able to fix nitrogen with both alfalfa (*Medicago sativa* L.) and the common bean [*Phaseolus vulgaris* (L) Savi], albeit at levels much lower than the usual microsymbionts of these hosts. *M. sativa* is usually nodulated by strains of *Ensifer* (ex *Sinorhizobium*) *meliloti* (Béna et al., 2005), but this bacterial species is known to be acid-sensitive and unable to survive in low pH soils (Garau et al., 2005; Reeve et al., 2006). *P. vulgaris* microsymbionts are most commonly species of *Rhizobium* (Eardly et al., 1985; reviewed in Graham, 2008). Because these two hosts were thought to be nodulated by distinctly different rhizobial genera, and the genes determining symbiotic host range in *Rhizobium* spp. were known to reside on plasmids (Rogel et al., 2011), it was thought that perhaps these Oregon isolates might represent recombinants of one species that had somehow acquired the genetic host-range determinants of the other. However, comparative sequence analysis of a partial segment of the 16S rRNA gene of OR191 revealed that this strain belonged to a distinct and previously unrecognized taxon (Eardly et al., 1992). In the same study it was also observed that OR191 was able to grow on strongly acidic agar media (pH 5.2), as did most of the *P. vulgaris* rhizobial symbionts examined. In contrast, *M. sativa* symbionts were unable to grow at pH 5.2 or below.

Strains sharing the unique host range, limited symbiotic efficiency, and acid-tolerance characteristics of OR191 were subsequently isolated from alfalfa growing at two other locations. In 1992, several “OR191-like” strains, including the strain T1155, were isolated from weakly acid soils in Ontario, Canada (Bromfield et al., 2001, 2010). Subsequently other OR191-like strains were isolated from moderately acid soils in Buenos Aires, Argentina (Del Papa et al., 1999). One of these strains, LPU83^T^, has now been proposed as the type strain for the new species *Rhizobium favelukesii* (Torres Tejerizo et al., 2016). The genome of LPU83^T^ (NZ_HG916852) has been established and consists of five replicons: the chromosome (4,195,305 bp); the chromid pLPU83d (1,932,030 bp); the accessory plasmids pLPU83a (151,687 bp) and pLPU83c (759,787 bp) and the symbiotic plasmid pLPU83b (ca. 531,535 bp) (Wibberg et al., 2014). pLPU83b carries all the nodulation and nitrogen fixation genes required to establish and maintain a nitrogen-fixing symbiosis (Wibberg et al., 2014).

OR191 and these other *R*. *favelukesii* strains thus represent a divergent lineage of acid-adapted strains within the genus *Rhizobium* that are able to fix N_2_ with both alfalfa and common bean, legume hosts that are each normally nodulated by different genera within the family Rhizobiaceae. Here we have sequenced the genome of *R. favelukesii* OR191, as part of the JGI 2010 GEBA-RNB project (Reeve et al., 2015; Seshadri et al., 2015). In this paper we provide an analysis of this genome and perform a comparison with the genomes of strains that are microsymbionts of alfalfa, or common bean, or of both. The genome properties of OR191 should provide insights into the diversification of the genus *Rhizobium*, the mechanisms involved in rhizobial acid-stress tolerance, and the roles that various genetic determinants play in the development of symbiotic relationships with two widely divergent legume hosts. In addition, due to the importance of exopolysaccharide (EPS) both for rhizobial symbiosis with legumes and for acid stress tolerance (Hawkins et al., 2017), we quantified the amount of EPS and substituents produced under neutral and acidic pH growth conditions, and determined its composition by NMR spectroscopy.

## Materials and Methods

### Bacterial Strains and Growth Conditions

Cultures of *R. faveluksii* OR191 were routinely sub-cultured on TYC solid medium (Howieson and Dilworth, 2016) incubated at 28°C for 3–4 days. For long-term maintenance, bacterial strains were grown in TYC broth and preserved in 20% glycerol at –80°C.

### Genomic DNA Preparation

*R. favelukesii* OR191 was streaked onto TYC solid medium and grown at 28°C for 3 days to obtain well grown, well separated colonies. A single colony was selected and used to inoculate 5 ml TYC broth medium. The culture was grown for 48 h on a gyratory shaker (200 rpm) at 28°C. Subsequently 1 ml was used to inoculate 60 ml TYC broth medium and grown on a gyratory shaker (200 rpm) at 28°C until an OD_600n*m*_ of 0.6 was reached. DNA was isolated from 60 ml of cells using a CTAB bacterial genomic DNA isolation method (Joint Genome Institute, 2022). Final concentration of the DNA was set to 0.5 mg ml^–1^.

### Genome Sequencing and Assembly

The draft genome of *Rhizobium favelukesii* OR191 was generated at the DOE Joint Genome Institute (JGI) using Illumina data (Bennett, 2004). An Illumina standard shotgun library was constructed and sequenced using the Illumina HiSeq 2000 platform which generated 17,712,488 reads totaling 2,657 Mbp. All raw Illumina sequence data were passed through DUK, a filtering program developed at JGI, which removes known Illumina sequencing and library preparation artifacts (Mingkun, L., Copeland, A. and Han, J., unpublished). The following steps were then performed for assembly: (1) filtered Illumina reads were assembled using Velvet (version 1.1.04) (Zerbino, 2010), (2) 1–3 Kbp simulated paired end reads were created from Velvet contigs using wgsim (Github and GitHub, 2011), (3) Illumina reads were assembled with simulated read pairs using Allpaths–LG (version r39750) (Gnerre et al., 2011). Parameters for the assembly steps were 1) Velvet: –v –s 51 –e 71 –i 2 –t 1 –f “-shortPaired -fastq $FASTQ” –o “-ins_length 250 -min_contig_lgth 500” for Velvet and 2) wgsim: -e 0 -1 76 -2 76 -r 0 -R 0 -X 0. The final draft assembly contained 240 contigs in 240 scaffolds. The total size of the assembly is 7.4 Mbp with an average of 350 × coverage of the genome.

### Genome Annotation

Genes were identified using Prodigal (Hyatt et al., 2010), as part of the DOE-JGI genome annotation pipeline (Mavromatis et al., 2009; Chen et al., 2013). The predicted CDSs were translated and used to search the National Center for Biotechnology Information (NCBI) non-redundant database, UniProt, TIGRFam, Pfam, KEGG, COG, and InterPro databases. The tRNAScanSE tool (Lowe and Eddy, 1997) was used to find tRNA genes, whereas ribosomal RNA genes were found by searches against models of the ribosomal RNA genes built from SILVA (Pruesse et al., 2007). Other non–coding RNAs such as the RNA components of the protein secretion complex and the RNase P were identified by searching the genome for the corresponding Rfam profiles using INFERNAL (Nawrocki and Eddy, 2013). Additional gene prediction analysis and manual functional annotation was performed within the IMG-Expert Review system (Markowitz et al., 2009) developed by the Joint Genome Institute, Berkeley, CA, United States.

### Phylogenetic Analysis

We assessed the phylogenetic position of *R. favelukesii* OR191, relative to other *Rhizobium* type and non-type strains, using a 1,296 bp internal region of the 16S rRNA gene. The *Azorhizobium caulinodans* ORS 571^T^ sequence was used as an outgroup. Phylogenetic analyses were performed using MEGA X (Kumar et al., 2018). The tree was built using the maximum likelihood method with the Tamura 3- parameter model (Tamura, 1992). A discrete Gamma distribution was used to model evolutionary rate differences among sites [5 categories (+ G, parameter = 0.3233)]. The rate variation model allowed for some sites to be evolutionarily invariable [( + I), 42.2407% sites]. Bootstrap analysis (Felsenstein, 1985) with 500 replicates was performed to assess the support of the clusters.

For phylogenetic analysis of the NodA proteins, where more than one *nodA* allele was present in the genome, we used the translated amino acid sequence of the *nodA* gene that was immediately upstream of *nodBC*. The evolutionary history was inferred by using the Maximum Likelihood method based on the Tamura 3-parameter model. The tree with the highest log likelihood (4204.9163) is shown. The percentage of trees in which the associated taxa clustered together is shown next to the branches. Initial tree(s) for the heuristic search were obtained automatically by applying Neighbor-Join and BioNJ algorithms to a matrix of pairwise distances estimated using the Maximum Composite Likelihood (MCL) approach, and then selecting the topology with superior log likelihood value. A discrete Gamma distribution was used to model evolutionary rate differences among sites [5 categories (+ G, parameter: 3.2381)]. The rate variation model, used for *nodA*, allowed for some sites to be evolutionarily invariable [( + I), 18.6224% sites]. The trees were drawn to scale, with branch lengths measured in the number of substitutions per site. The analysis involved 41 nucleotide sequences for *nodA*. All positions containing gaps and missing data were eliminated. In the final dataset there were a total of 588. Evolutionary analyses were conducted in MEGA X (113).

Phylogenetic analysis and evolutionary history of the NifH proteins was inferred by using the Maximum Likelihood method and JTT matrix-based model (Jones et al., 1992). The tree with the highest log likelihood (–8402.67) is shown. The percentage of trees in which the associated taxa clustered together is shown next to the branches. Initial tree(s) for the heuristic search were obtained automatically by applying Neighbor-Join and BioNJ algorithms to a matrix of pairwise distances estimated using the JTT model, and then selecting the topology with superior log likelihood value. A discrete Gamma distribution was used to model evolutionary rate differences among sites [5 categories (+ *G*, parameter = 2.4848)]. The rate variation model allowed for some sites to be evolutionarily invariable [( + *I*), 14.51% sites]. The tree is drawn to scale, with branch lengths measured in the number of substitutions per site. This analysis involved 38 amino acid sequences; *Rhizobium mesoamericanum* STM3655 and *Ensifer meliloti* AK53 were excluded from the analysis due to a lack of sequencing information. There were a total of 572 positions in the final dataset. Evolutionary analyses were conducted in MEGA X (Kumar et al., 2018).

### Genome Average Nucleotide Identity Pairwise Comparisons

gANI comparisons were performed using the IMG gANI analysis tool (Chen et al., 2021).

### Exopolysaccharide Production

Cells of OR191 and *Ensifer medicae* WSM419 were grown to mid-exponential phase at pH 7.0 in JMM minimal salts media (Howieson and Dilworth, 2016), washed in JMM at pH 7.0 and then resuspended in JMM at either pH 7 or 5.8 to an OD_600 *nm*_ of approximately 0.01 for pH 7.0 media (100 ml) and 0.25 for pH 5.8 media (100 ml). Cells were incubated at 28°C with shaking at 200 rpm for 4 days. Cells were then removed by centrifugation (10 min at 7,000 *g*) and EPS was precipitated from the supernatant by adding 0.1 vol. of a 2% hexadecyltrimethyl-ammonium bromide solution stored at 28°C. EPS was pelleted (10 min at 7,000 *g*) and redissolved in 10% (w/v) NaCl. The EPS was precipitated by adding 2 volumes of acetone, pelleted (10 min at 7,000 *g*), redissolved in sterile water and dialyzed in water. EPS was lyophilized, resuspended in D_2_O (99.96%.), lyophilized again, and dissolved in D_2_O (99.96%.) to a concentration of 15 mg ml^–1^. ^1^H-NMR spectra were recorded on a Varian 400-MR spectrometer at 80°C. A 30 μl aliquot of a 2% (w/v) solution of the succinoglycan-binding dye Calcofluor (Fluorescent brightener 28, Sigma) was added to 1 ml samples containing 7.5 mg EPS in saline [0.89% (w/v)] to visualize fluorescence at a wavelength of 365 nm.

## Results and Discussion

### OR191 Genome Characteristics, Properties and Phylogenetic Placement

OR191 is a rod shaped isolate that is fast growing and forms typically mucoid colonies on conventional media used to isolate rhizobia (Howieson and Dilworth, 2016; Supplementary Figure 1). Flagella are not present in the transmission electron micrograph of OR191 (Supplementary Figure 1B), which is consistent with the description of the *R. favelukesii* type strain LPU83^T^ as non-motile (Torres Tejerizo et al., 2016). The classification, general features and genome sequencing project information for OR191 are provided in Supplementary Table 1, in accordance with the minimum information about a genome sequence (MIGS) recommendations (Field et al., 2008) published by the Genomic Standards Consortium (Field et al., 2011).

The draft genome of strain OR191 was generated at the DOE Joint Genome Institute (JGI) using Illumina data (see “Materials and Methods” section). A summary of the genome project features for OR191 is shown in Table 1. The genome is 7,368,160 bp with 59.66% GC content and is comprised of 240 scaffolds. From a total of 7,704 genes, 7,617 were protein encoding and 87 RNA-only encoding genes. The majority of genes (72.12%) were assigned a putative function whilst the remaining genes were annotated as hypothetical. The distribution of genes into COGs functional categories is presented in Supplementary Table 2.

**TABLE 1:** Genome statistics for *Rhizobium favelukesii* strain OR191.

Attribute	Value	% of total
Genome size (bp)	7,368,160	100.00
DNA coding (bp)	6,276,762	85.19
DNA G+C (bp)	4,395,536	59.66
DNA scaffolds	240	100.00
Total genes	7,704	100.00
Protein-coding genes	7,617	98.87
RNA genes	87	1.13
Pseudo genes	0	0.00
Genes in internal clusters	1,605	20.83
Genes with function prediction	5,617	72.91
Genes assigned to COGs	4,702	61.03
Genes with Pfam domains	5,827	75.64
Genes with signal peptides	576	7.48
Genes with transmembrane proteins	1,585	20.57
CRISPR repeats	1	N/A

Many of the features of OR191 are indistinguishable from those of *R. favelukesii* LPU83^T^ and T1155, including host range, *nod* and *nif* genes, insertion-sequence hybridization profiles, PCR-based genomic fingerprints, and plasmid profiles (Eardly et al., 1985, 1992; Del Papa et al., 1999; Segundo et al., 1999; Wegener et al., 2001; Hou et al., 2009; Bromfield et al., 2010; Wibberg et al., 2014; Table 2). Consistent with the plasmid profiles, we identified genes encoding homologs of the Rep proteins (required for the replication and stable maintenance of plasmid replicons) in the OR191 genome that shared 100% identity with those present in the *R. favelukesii* pLPU83a, pLPU83b, pLPU83c, and pLPU83d plasmids, suggesting that both *R. favelukesii* strains possess the same number of replicons. Furthermore, the OR191 genome contains two separate regions that encode proteins required for DNA transfer replication and mating pair formation, which share very high identity with homologs in the characterized plasmids pLPU83a (which is conjugative) and pLPU83b (which is mobilizable) (Torres Tejerizo et al., 2010, 2014). We also found genes encoding a separate conjugative system in OR191 that shares very high identity with a conjugative system that is present on the pLPU83d chromid. LPU83^T^ shares its lipopolysaccharide profile with OR191 (Wegener et al., 2001), while T1155 shares its phage-resistance type with OR191 (Bromfield et al., 2010). Furthermore, the DNA-DNA hybridization values between strains LPU83^T^ and OR191 were greater than 84%, indicating that these strains are members of the same species (Torres Tejerizo et al., 2016). DNA alignments of OR191 essential “housekeeping” gene sequences (including 16S rRNA, 23S rRNA, *recA*, and *atpD* genes) to the corresponding genes of LPU83^T^, T1155 and the closely related *Rhizobium tibeticum* CGMCC 1.7071^T^ gave 100% sequence identity of OR191 genes to those of LPU83^T^ and from 98.83 to 100% sequence identity to those of T1155 or CGMCC 1.7071^T^ (Table 2). In the 16S rRNA phylogenetic tree, OR191 is in a clade with *R. grahamii* CCGE 502^T^, *R. favelukesii* strains and *R. tibeticum* CGMCC 1.7071^T^ (Supplementary Figure 2).

**TABLE 2:** A descriptive summary of the phenotypic and genotypic characteristics of *Rhizobium favelukesii* OR191 compared to three other closely related *Rhizobium* strains that have the same extended host range as OR191.

Descriptor	Genus/species				
	*Rhizobium favelukesii*	*Rhizobium favelukesii*	*Rhizobium favelukesii*	*Rhizobium tibeticum*	References
Strain	OR191	LPU83^T^	T1155	CGMCC 1.7071^T^	Eardly et al., 1985; Del Papa et al., 1999; Hou et al., 2009; Bromfield et al., 2010
Origin	Oregon, United States	Buenos Aires, Argentina	Ontario, Canada	Tibet, China	Eardly et al., 1985; Del Papa et al., 1999; Hou et al., 2009; Bromfield et al., 2010
**Nodulation^a^**					
*Medicago sativa*	**+**	**+**	**+**	**+**	Eardly et al., 1985; Del Papa et al., 1999; Hou et al., 2009; Bromfield et al., 2010
*Phaseolus vulgaris*	**+**	**+**	**+**	**+**	
**Symbiotic effectiveness^b^**					
*Medicago sativa*	31	76	64	–	Segundo et al., 1999; Bromfield et al., 2010
*Phaseolus vulgaris*	78	–	43	–	Bromfield et al., 2010
**Profiles**					
IS/nod genotype	OR191	= OR191	= OR191	–	Wegener et al., 2001; Bromfield et al., 2010
LPS profile	OR191	= OR191	–	–	Wegener et al., 2001
Plasmid profile	OR191	= OR191	= OR191	–	Del Papa et al., 1999; Bromfield et al., 2010
**PCR fingerprint pattern**					
MBO REP	OR191	= OR191	–	–	Del Papa et al., 1999
ERIC	OR191	= OR191	–	–	Wegener et al., 2001
**% Sequence similarity to genes in the OR191 genome**					
16S rRNA	100	100	100	99.9	This study
23S rRNA	100	96.7	99.9*	99.8	This study
*recA*	100	100	100*	99.4	This study
*atpD*	100	100	98.7*	98.8	This study

*^a^A plus (+) sign denotes that nodule numbers on the respective hosts were similar to the numbers observed for the control strains in the respective studies.*

*^b^Relative symbiotic effectiveness (%) is based on strain shoot dry weight production relative to that observed for effective control strains.*

*Partial sequence.

gANI pairwise comparisons were also calculated for *Rhizobium* genomes deposited in IMG that were most closely related to OR191 in the 16S rRNA phylogenetic tree. The genome of *R. favelukesii* T1155 could not be used in this analysis as it has not yet been sequenced. gANI values for the most closely related strains are shown in Table 3; scores over the defined species affiliation cut-off value of > 96.5 gANI (Varghese et al., 2015) are shown in bold font. The gANI pairwise comparisons, along with other previously presented phenotypic and genotypic data, confirmed that OR191 is conspecific with *R. favelukesii* LPU83^T^. The gANI results also suggest that *R. tibeticum* CGMCC 1.7071^T^ belongs to the same species as LPU83^T^ and OR191. However, the DND-DNA hybridization values obtained by Torres Tejerizo et al. (2016) indicate that *R. favelukesii* and *R. tibeticum* strains are separate species; moreover, *R. tibeticum* strain CGMCC 1.7071^T^ shared lower sequence identities of the *recA*, *atpD*, and *rpoB* genes than the *R. favelukesii* strains and differed in its plasmid profile, fatty acid profile and ability to metabolize several sole carbon substrates.

**TABLE 3:** Pairwise comparisons of gANI values^a^ of *Rhizobium favelukesii* OR191 (shaded) to selected *Rhizobium* strains in IMG.

Strain	Gold ID Gp	Genome size (bp)	LPU83^T^*	OR191	CGMCC 1.7071^T^	CCGE 502^T^	STM3625	STM6155
*R. favelukesii* LPU83^T^	0101044	7,569,648		**99.99**	**97.59**	85.05	85.63	85.66
*R. favelukesii* OR191	0009662	7,368,160	**99.99**		96.59	85.04	85.62	85.66
*R. tibeticum* CGMCC 1.7071^T^	0120274	7,065,782	**96.58**	**96.59**		85.14	85.59	85.72
*R. grahamii* CCGE 502^T^	0010596	7,146,037	85.08	85.05	85.13		85.26	85.39
*R. mesoamericanum* STM3625	0023271	6,453,427	85.60	85.59	85.59	85.27		96.90
*R. mesoamericanum* STM6155	0009783	6,927,906	85.65	85.66	85.72	85.40	96.91	

*^a^gANI values were calculated in pairwise comparisons using the IMG gANI algorithm. gANI values above the threshold species cut-off are shown in bold.*

**TABLE 4:** Relative amounts of substitution on EPS from *Rhizobium favelukesii* OR191 and *Ensifer medicae* WSM419 grown at pH 7.0 and 5.8.

OR191							

			**Ratios**				
**pH**	**Substituent**	Relative amount*	**3-OH butyrate methylene/acetyl**	**3-OH butyrate methyl/pyruvyl**	**3-OH butyrate methylene/acetyl**	**3-OH butyrate methyl/pyruvyl**	**Acetyl/pyruvyl**
7.0	Acetyl	8.71					0.56
	Pyruvyl	15.65					
	3-OH butyrate methylene	1.50	0.17	0.10			
	3-OH butyrate methyl	2.58	0.30	0.16			
5.8	Acetyl	9.48					0.65
	Pyruvyl	14.67					
	3-OH butyrate methylene	2.90	0.31	0.20			
	3-OH butyrate methyl	4.66	0.49	0.32			

**WSM419**							

			**Ratios**				
**pH**	**Substituent**	Relative amount*	**Succinyl/** **acetyl**	**Succinyl/** **pyruvyl**			**Acetyl/** **pyruvyl**

7.0	Acetyl	23.74					0.99
	Pyruvyl	23.97					
	Succinyl	9.09	0.38	0.43			
5.8	Acetyl	9.20					1.03
	Pyruvyl	8.95					
	Succinyl	9.43	1.03	1.05			

* Relative amounts of substituents have been normalized to 100 sugar “units” based on integrations of ^1^H NMR peaks for the chemical shifts indicated in the Methods.

### Whole Genome Comparisons of *Rhizobium favelukesii* Strains

The genes of the sequenced *R. favelukesii* strains OR191 and LPU83^T^ were compared, using the default parameters of 30% protein identity and E-value of 1e-5 in the JGI IMG phylogenetic profiler. Of a total of 7,704 protein coding genes in OR191, 7,449 genes were found to be in common with LPU83^T^. The remaining 255 genes were unique to OR191, with nearly half annotated as hypothetical (128 genes representing 50.20%). These hypothetical genes included extensive prophage loci clustered on scaffold 24.25. By using the prophage predicting tool PHASTER (Arndt et al., 2016) these loci were shown to encode a complete resident prophage. Whole genome analysis of OR191 found four additional resident prophages present on scaffolds 4.5, 7.8, 84.85, and 91.92 (Supplementary Figure 3), in addition to the unique prophage on scaffold 24.25.

### Identification and Comparison of Genes Involved in Acid Tolerance in OR191

Strain OR191 was isolated from alfalfa growing in moderately acidic soil (Eardly et al., 1985) and can grow in laboratory culture at pH 5.2 (Eardly et al., 1992). Most *P. vulgaris Rhizobium* microsymbionts can also grow in laboratory culture at pH 5.2 (Eardly et al., 1992). In contrast, the *E. meliloti* strains that are the usual microsymbionts of *M. sativa* are known to be particularly acid-sensitive in comparison to other rhizobial species, and in laboratory culture fail to grow below pH 5.6 (Graham and Parker, 1964; Eardly et al., 1992; Garau et al., 2005). In order to gain insights into the acid tolerance of OR191, we examined the genome for the presence of loci known to be important for the pH adaptation of very acid tolerant strains (such as *Rhizobium tropici* CIAT899^T^) and to less acid tolerant but well characterized strains (such as *Ensifer meliloti* 1021 and *E. medicae* WSM419). Although less acid-tolerant than CIAT899^T^, WSM419 is the dominant nodule occupant of *Medicago* species growing in moderately acidic soils in Sardinia and Greece (Garau et al., 2005).

Most of the genes found to be important for acid tolerance in *Rhizobium* strains had orthologs in WSM419 and *Ensifer meliloti* 1021 (Supplementary Table 3), including the most acid-activated genes discovered so far, *lpiA* and *acvB* (Reeve et al., 2006), encoding lysyl-phosphatidylglycerol synthase and lysyl-phosphatidylglycerol hydrolase, respectively, and required for modulation of lysyl-phosphatidylglycerol homeostasis (Sohlenkamp et al., 2007). However, the *fsrR*, *tcrA*, and *tcsA* regulatory system, which is implicated in the strong acid induction of the *lpiA-acvB* operon in WSM419, is not present in OR191 or in species other than *E. medicae*, indicating that an unknown regulatory system governs acid induction of these genes in other rhizobia (Reeve et al., 2006; Tian et al., 2017). In OR191 and several other *Rhizobium* strains, the *lpiA-acvB* operon is immediately downstream of a gene encoding a small-conductance mechanosensitive channel, a membrane protein that is important for bacterial survival (reviewed in Naismith and Booth, 2012). The *olsC* gene, which has been identified as being important for the production of hydroxylated ornithine lipid species that increase stress tolerance in CIAT899^T^ (Rojas-Jiménez et al., 2005; Vences-Guzmán et al., 2011), was present in both the highly acid tolerant strain *R. tropici* CIAT899^T^ and in *R. favelukesii* strains but was absent from *E. medicae* WSM419 and *E. meliloti* 1021. This accords with proteomic and transcriptomic studies of *R. favelukesii* LPU83^T^ that revealed changes in proteins and genes associated with lipid metabolism involving the cell envelope in response to acid stress, however, these studies also suggested that some acid-responsive regulatory systems differ in LPU83^T^ compared with *Ensifer* strains (Nilsson et al., 2019, 2020).

### Comparisons of the OR191 Genome With Other Microsymbionts of Medicago and/or *Phaseolus vulgaris*

*R. favelukesii* OR191 nodulates and fixes nitrogen with both *M. sativa* and *P. vulgaris*. To identify features within the OR191 genome that enable it to be a microsymbiont of both these hosts, the IMG online database was used to compare relevant sequenced genomes of microsymbionts of *Medicago* species, or of common bean, or of both (strains listed in Supplementary Table 4).

We examined these genomes for genes with known roles in rhizobial symbiotic interactions. In addition to meeting or exceeding the cut-off values of > 30% protein identity over > 75% protein coverage, OR191 *nod*, *nif* and *fix* (*cco*) homologs were validated as orthologous if they were present within the neighborhood of symbiotic gene clusters. A summary of the identified OR191 genes with characterized roles in symbiotic interactions and their phylogenetic profile in microsymbionts of *Medicago* and *P. vulgaris* is provided in Supplementary Table 5.

### Overview of the Nod Genes in OR191

Symbiosis between legumes and rhizobia is initiated by a molecular dialogue, in which the host secretes flavonoid signals from the root cells, triggering expression of the rhizobial nodulation (*nod, noe, and nol*) genes and production of lipochitoligosaccharide Nod factors, which are required for infection of the host and nodule organogenesis. Expression of the nodulation genes is controlled by the regulatory protein NodD. In addition to the Nod factor core biosynthetic genes (*nodABC*) and export genes (*nodIJ*), rhizobial genomes contain accessory nodulation genes, which encode moieties that decorate the basic Nod factor and act as specific host determinants (Perret et al., 2000).

The core and accessory nodulation genes of OR191 are distributed on four scaffolds (Supplementary Figure 4) and share 99–100% sequence identity with those of *R. favelukesii* LPU83^T^. While all rhizobial genomes contained the core nodulation genes *nodABCDIJ*, there were differences in the accessory nodulation genes present within *Medicago* and *P. vulgaris* microsymbionts.

### OR191 Nod Genes That Are Common to *Medicago* Microsymbionts

In addition to the core *nodABCIJ* genes, all *Medicago* microsymbiont genomes (*E. meliloti* strains + OR191, LPU83^T^, *R. tibeticum* CGMCC 1.7071^T^ and *R. mongolense* USDA 1844^T^) contained a specific set of accessory *nod* genes. All *Medicago* microsymbiont genomes harbored three *nodD* alleles, which in *E. meliloti* appear to be important in optimizing symbiotic interactions with *Medicago* hosts (Honma et al., 1990; Van Berkum et al., 1998). The OR191 *nodD* genes are most closely related to orthologs in LPU83^T^ and the *Medicago*-nodulating *Rhizobium* strains *R. tibeticum* CGMCC 1.7071^T^ and *R. mongolense* USDA 1844^T^. All *Medicago* microsymbiont genomes also contained the *nodL* and *nodHPQ* genes that code for synthesis of acetylated and sulfated Nod factors, respectively, which in *Ensifer* strains are required for symbiotic interaction with *Medicago* hosts (Lerouge et al., 1990). The Nod factors of *R. favelukesii* LPU83^T^ include species with trimeric, tetrameric, and pentameric chitin backbones that contain unsaturated acyl chains and may be sulfated or methylated, however, sulfated Nod factors do not appear to be required for this strain to nodulate alfalfa, as mutation of *nodH* delayed, but did not abolish, nodulation of this host (Del Papa et al., 2007; Torres Tejerizo et al., 2011). Both LPU83^T^ and OR191 contained the flavonoid-inducible *nodL*, *noeA*, and *noeB* operon, which is required for effective nodulation of *Medicago* sp. (Ardourel et al., 1995). NoeA and NoeB are hypothesized to modify the Nod factor for accurate recognition by *Medicago* spp., allowing the microsymbiont to successfully infect the host.

The *nodEF* genes, which are required for production of Nod factors with α, β-unsaturated acyl chains (Dénarié et al., 1996), were also present in all *Medicago* microsymbiont genomes. NodE has homology to β-ketoacyl synthases, while NodF is homologous to acyl carrier proteins (Demont et al., 1993). The *nodG* gene, encoding a 3-oxoacyl-acyl carrier protein reductase, was identified in all *Medicago*-nodulating *E. meliloti* genomes. NodG has a postulated role in fatty acid synthesis in the *M. sativa* microsymbiont strain *E. meliloti* 1021 (Mao et al., 2016), but is not required for synthesis of the characteristic α, β-unsaturated acyl chains of the Nod factors (Debellé et al., 2001). Although *nodG* is not essential for nodulation of *M. sativa*, it does increase nodulation efficiency (Mao et al., 2016). In OR191, the gene immediately upstream of *nodP* has sequence similarity to the characterized *nodG* of *E. meliloti* but encodes a truncated protein of 55 amino acids instead of the 245 amino acids found for *E. meliloti* NodG. The lack of a functional NodG might partially explain OR191’s less efficient nodulation performance on *M*. *sativa*, in comparison to 1021. *R. favelukesii* LPU83^T^ also contains a truncated version of *nodG* (Torres Tejerizo et al., 2011). We identified similarly truncated *nodG* genes in the same gene neighborhoods within the genomes of *R. tibeticum* CGMCC 1.7071^T^ and *R. mongolense* USDA 1844^T^, indicating that *nodG* pseudogenes may be a feature of these strains. The *nolFG* genes, encoding an RND-type efflux pump, are also characteristic of *Medicago-*nodulating *Ensifer* strains (Baev et al., 1991) and disruption of this region leads to delayed nodulation of alfalfa (Baev et al., 1991).

The OR191 *nodABC, nodEF, nodL, nodHPQ*, and *nolFG* genes are most closely related to those of LPU83^T^, CGMCC 1.7071^T^ and USDA 1844^T^, and then to orthologs in *Ensifer* strains that nodulate *Medicago.* On the basis of its *nod* genes and symbiotic phenotype, Rogel et al. (2011) have included OR191 in the symbiovar orientale (Silva et al., 2005; Rogel et al., 2011), along with *R. favelukesii* LPU83^T^, *R. gallicum* CCBAU 01083, *R. mongolense* USDA 1844^T^, and *R. tibeticum* CGMCC 1.7071^T^ (Van Berkum et al., 1998; Zhao et al., 2008; Hou et al., 2009; Torres Tejerizo et al., 2016).

### OR191 Nod Genes Common to *Phaseolus vulgaris* Microsymbionts

A comparison of OR191 to other *P. vulgaris* microsymbionts revealed that the only *nod* genes they have in common are the core *nodABCDIJ* genes. Both the types of *nod* genes and the gene arrangements varied considerably in the *P. vulgaris* microsymbionts; for example, from two to six *nodD* alleles and from one to three *nodA* alleles were found and *nodHPQ* and *nodEF* clusters were present in some genomes but not others. Similarly, *nodU*, *nodZ, noeI, noeT*, and *nolL* genes were variably present or absent. However, all the *P. vulgaris* microsymbionts except OR191, LPU83^T^, *R. tibeticum* CGMCC 1.7071^T^ and *R. mongolense* USDA 1844^T^ contained *nodS*, which encodes an S-adenosyl-L-methionine (SAM)-dependent N-methyltransferase. Notably, *nodS* is absent from the genomes of all *Medicago* microsymbionts. A *nodS*-dependent N-methylation on the non-reducing end of the Nod Factor has been shown to be essential for the nodulation of *P*. *vulgaris* by the *Rhizobium tropici* strain CIAT 899^T^ (Jabbouri et al., 1995). Although the *Medicago* microsymbionts (including the *R. favelukesii*, *R. tibeticum*, and *R. mongolense* strains) lack *nodS*, they contain the *nodL* gene required for production of *O*-acetylated Nod factors. NodL acetylation of the Nod factor functionally prevents the NodS-dependent transfer of the *N*-methyl group substituent, however, it has been proposed that NodL-dependent *O*-acetylation of the Nod factor can compensate for the lack of this *N*-methyl group (Waelkens et al., 1995; Lopez-Lara et al., 2001). Nodulation of *P*. *vulgaris* would appear to require either an acetylated or a methylated Nod factor, which can be accomplished by NodL or NodS, respectively.

The *nodEF* genes were found in *R. favelukesii*, *R. tibeticum*, and *R. mongolense*, *Ensifer fredii* GR64, *E. meliloti* GVPV12, *Ensifer* sp. strains 4H41 and BR816 and strains closely related to *R. tropici* CIAT 899^T^, but not in the remaining *P. vulgaris*-nodulating strains. Although the characterized strain *R. tropici* CIAT 899^T^ contains *nodEF*, it does not produce Nod factors with α,β-unsaturated acyl chains (Ormeño-Orrillo et al., 2012). This can be related to the inability of *R. tropici* NodA to transfer unsaturated acyl chains to the Nod factor, whereas NodA of *E. meliloti Medicago* microsymbionts specifies the N-acylation of the Nod factor by an unsaturated or hydroxylated fatty acid (Debellé et al., 1996). However, the CIAT 899^T^ *hsnT*, *nodF*, and *nodE* genes, which are in an operon with *nodA2* in this strain, are important for the biosynthesis and specific decoration of the Nod Factors, with resulting impact on host specificity and symbiotic performance in some legume species (Gomes et al., 2019).

**FIGURE 1:**
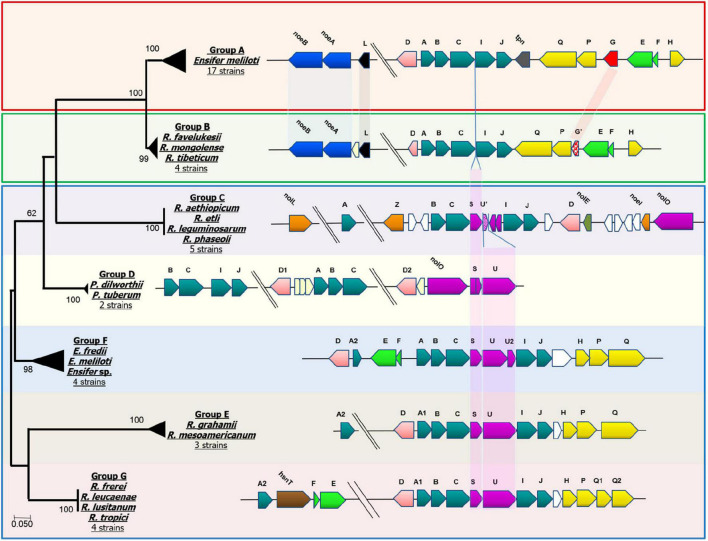
NodA phylogenetic distribution and comparison of gene neighborhoods. Comprehensive list of strains in each group shown and not shown can be found in Supplementary Table 4. Group A: *Ensifer meliloti* 1021, *Ensifer meliloti* 2011, *Ensifer meliloti* 5A14, *Ensifer meliloti* AE608H, *Ensifer meliloti* AK58, *Ensifer meliloti* AK83, *Ensifer meliloti* BL225C, *Ensifer meliloti* BO21CC, *Ensifer meliloti* CCNWSX0020, *Ensifer meliloti* CIAM1775, *Ensifer meliloti* GR4, *Ensifer meliloti* Mlalz-1, *Ensifer meliloti* MVII-I, *Ensifer meliloti* Rm41, *Ensifer meliloti* RRI128, *Ensifer meliloti* SM11 and *Ensifer meliloti* WSM1022. Group B: *Rhizobium favelukesii* OR191, *Rhizobium favelukesii* LPU83, *Rhizobium mongolense* USDA 1844, *Rhizobium tibeticum* CGMCC 1.7071. Group C: *Rhizobium aethiopicum* HBR26, *Rhizobium etli* CFN 42, *Rhizobium leguminosarum* s.s. 4292, *Rhizobium leguminosarum* genospecies K FA23, *Rhizobium phaseoli* CIAT 652. Group D: *Paraburkholderia dilworthii* WSM3556 and *Paraburkholderia tuberum* WSM4176. Group E: *Rhizobium mesoamericanum* STM 3625 and *Rhizobium mesoamericanum* STM6155. Group F: *Ensifer fredii* GR64, *Ensifer* sp. 4H41 and *Ensifer meliloti* GVPV12. Group G: *Rhizobium leucaenae* USDA 9039, *Rhizobium lusitanum* P1-7 and *Rhizobium tropici* CIAT899.

Because the transfer of unsaturated fatty acid chains to the Nod factor backbone requires a specific NodA protein (Debellé et al., 1996), we performed a comparative sequence analysis of the NodA proteins of the *Medicago* and *P. vulgaris* microsymbionts. The resulting phylogenetic tree grouped NodA into eight clusters (Group A—G, Figure 1) and showed that Cluster B (NodA of *Medicago*-nodulating *R. favelukesii*, *R tibeticum, and R mongolense* strains) formed a sister group to Cluster A of the *Medicago*-nodulating *E. meliloti* strains. This illustrates that even though *E. meliloti* 4H41 and GVPV12, *Ensifer* sp. BR816 and strains closely related to *R. tropici* CIAT 899^T^ contain *nodEF* and therefore are potentially able to produce α, β-unsaturated acyl chains, their NodA is phylogenetically distinct from the NodA of *Medicago*-nodulating strains that can transfer unsaturated acyl chains to the Nod factor.

By mapping *nod* gene neighborhoods to the NodA phylogenetic tree, we identified that particular arrangements of *nod* genes are associated with each NodA group (Figure 1). The figure illustrates the high degree of synteny between the *nod* genes of the *Medicago*-nodulating *Ensifer* strains (Group A) and the *R. favelukesii*, *R tibeticum and R mongolense* strains (Group B). *R. tibeticum* CGMCC 1.7071^T^ is the microsymbiont of *Medicago ruthenica*, while *R. mongolense* USDA 1844^T^ is the microsymbiont of *Medicago archiducis-nicolai* (Van Berkum et al., 1998; Hou et al., 2009). Both these *Medicago* species belong to the basal section Platycarpae and are distributed in Siberia, Mongolia, Tibet and northern China, which is far to the northeast of alfalfa’s center of origin (Small, 2010; Steele et al., 2010), suggesting that there may have been a change of microsymbiont in *Medicago* species, due to either biogeographical factors or a change in host preference. As noted previously, all *P. vulgaris*-nodulating strains (Group B—G) have either *nodS* or *nodL* in their genomes. In the strains belonging to Group D to G, *nodSU* is consistently located between *nodC* and *nodIJ.* The truncated *nodU* seen in the *P. vulgaris*-nodulating strains of Group C would appear to have arisen from an ancestral *nodSU* form. In the *Medicago*-nodulating strains belonging to Group A and B, the deletion of *nodSU* would account for *nodABCIJ* being adjacent to each other.

### Comparison of Nif and Fix Genes in *Medicago* and *Phaseolus vulgaris* Microsymbionts

**FIGURE 2:**
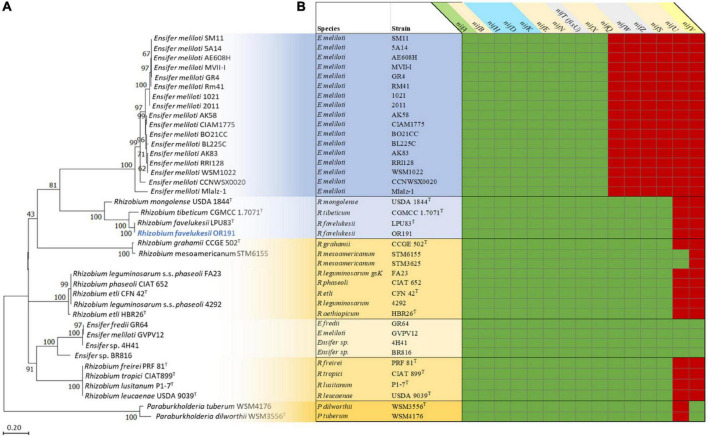
Analysis of the nitrogen fixation genes and proteins in *Medicago* and *Phaseolus vulgaris* microsymbionts (Supplementary Table 4). Phylogenetic analysis of the NifH proteins **(A)** and a map of *nif* genes present (green) or absent (red) in the genomes of the microsymbionts **(B)**. All microsymbionts shaded in blue nodulate and fix with *Medicago* sp. Strains shaded in yellow nodulate and fix with *Phaseolus vulgaris*. *Rhizobium favelukesii* LPU83, *Rhizobium mongolense* USDA 1844, *Rhizobium tibeticum* CGMCC 1.7071 can nodulate both *Medicago* spp. and *Phaseolus vulgaris*.

The rhizobial *nif, fix* and *fdx* genes are required for the stepwise reduction of dinitrogen gas and incorporation of fixed N (Fischer, 1994). Whereas the characterized rhizobial diazotroph *Azorhizobium caulinodans* ORS 571^T^ has 15 *nif* genes, most rhizobia are unable to fix nitrogen *ex planta* and their genomes contain fewer than 15 *nif* genes (Masson-Boivin et al., 2009). We identified 13 *nif* genes in OR191 (Supplementary Figure 4 and Supplementary Table 5). The genes and the associated processes that they are involved in are: *nifA* (regulation); *nifH* (dinitrogenase reductase); *nifDK* (α and β subunits of dinitrogenase); *nifB* (synthesis of the iron-sulfur-containing precursor of the FeMo-co); *nifEN* (assembly of the FeMo-co); *nifQSX* (FeMo-co biosynthesis), *nifZ* (nitrogenase maturation) and *fixU*/*nifTnifW* (nitrogen fixation accessory and stabilization proteins, respectively). OR191 lacked genes encoding NifU (iron-sulfur cluster scaffolding protein) and NifV (homocitrate synthase). The OR191 genome also contained genes required for production of ferredoxins (*fdxBN*), putative electron transfer proteins (*fixABCX*), the specific high-affinity *cbb3*-type cytochrome c oxidase (*fixNOQP*), a Cu^2+^-exporting ATPase (*fixI*), electron transfer components (*fixGHS*) and a putative FNR/CRP [fumarate and nitrate reductase regulator protein and cyclic AMP (cAMP) receptor protein] transcriptional regulator (*fixK*) (Supplementary Table 5). The OR191 *nif* and *fix* genes are most closely related to those of LPU83^T^, CGMCC 1.7071^T^ and USDA 1844^T^ and then to those of *Rhizobium* and *Ensifer* strains that nodulate *P. vulgaris*, rather than to the *nif* and *fix* genes of *Medicago*-nodulating *Ensifer* strain. Phylogenetic analysis of NifH and NifA shows that OR191, LPU83^T^, CGMCC 1.7071^T^, and USDA 1844^T^ form a clade in comparison to other microsymbiont strains analyzed in this study (Figure 2A).

Furthermore, a comparison of the *nif* genes present in the microsymbionts of *Medicago* spp. and *P. vulgaris* showed that all genomes contained the nitrogenase genes *nifABHDKENX* and *fixU/nifT* or *nifT*-like genes (encoding the specific NifT pfam06988), *fdxBN* and *fixABCX* genes (Figure 2B). All strains contained *fixGHIS* and *fixNOQP*, apart from the *Paraburkholderi*a microsymbionts (previously reported in De Meyer et al., 2016). The most striking differences between the suite of *nif* genes found in the *Medicago* and *P. vulgaris* rhizobia was that *Ensifer* strains that are *Medicago* microsymbionts all lacked *nifQWZSUV*, whereas strains that nodulate and fix with *P. vulgaris* all contained *nifQWZS.* All the *Rhizobium* strains contained *nifS* but lacked *nifU* and *nifV*. *Ensifer* strains that were microsymbionts of *P. vulgaris* (*E. fredii* GR64, *E meliloti* GVPV12 and *Ensifer* sp. 4H41 and BR816) all contained *nifU* and *nifV*, in addition to the 13 *nif* genes identified in OR191. The *nifU* gene was absent from the *Paraburkholderia* strains, but as rhizobial *Paraburkholderia* strains are able to fix N *ex planta* (Elliott et al., 2007), *nifU* may be replaced by an unidentified gene encoding a functionally equivalent protein.

### Comparisons of Other Symbiotically Relevant Genes in *Medicago* and *Phaseolus vulgaris* Microsymbionts

#### Succinoglycan Biosynthesis Genes

Other known rhizobial genes that are required for symbiotic interactions with legumes include those responsible for production of exopolysaccharides, which play an important role in the primary stage of infection by suppressing the plant defense response, as well as contributing to acidic pH tolerance (Skorupska et al., 2006; Hawkins et al., 2017). In the alfalfa-*E. meliloti* symbiosis, the specific exopolysaccharide succinoglycan (EPS I) is required for the formation of infection threads in *Medicago* root hairs and is necessary to prevent the expression of plant defense response genes (Jones and Walker, 2008), although the lack of EPS I may be overcome to some extent by the production of galactoglucan (EPS II) or capsular polysaccharide (Pellock et al., 2000). In the characterized strain *E. meliloti* 1021, the *exo* genes required for succinoglycan biosynthesis (*exoBZQFYXUVWTIHKLAMONP*) form a cluster on the pSymB of this strain (Finan et al., 2001; Janczarek, 2011).

Within the OR191 genome, we identified homologs of all these *exo* genes (Supplementary Table 5) except for *exoI* (the OR191 *exoI*-like gene, encoding a periplasmic endonuclease, shared greater sequence identity with paralogs of *exoI*-like genes within the 1021 genome). The *exoB* and *exoN* genes, encoding nucleotide sugar precursors, were located on scaffolds 2.3 and 77.78, respectively. The *expR* gene, encoding a LuxR family transcriptional regulator that regulates production of both EPS I and EPS II, was also located around 60 kbp downstream of *exoB* on scaffold 2.3. The remaining *exo* genes were in two separate clusters, with the nucleotide sequence of each cluster being highly conserved and nearly identical (>99% over the entire length) to corresponding *exo* clusters in *R. favelukesii* LPU83^T^. The *exo* genes of LPU83^T^ have been characterized; one cluster is on the chromosome and one cluster is located on the plasmid pLPU83a between two inverted repeat regions, suggesting an integration of DNA from another organism (Castellani et al., 2019, 2021). In OR191, Cluster I on scaffold 50.51 contained *exoZQFYXUWHKAMOP* and shared DNA sequence similarity of 99–100% with the *exo* cluster on the LPU83^T^ chromosome (NCBI accession number HG916852). The *R. favelukesii* Cluster I *exo* genes shared greatest synteny and sequence identity with *exo* genes of the *Rhizobium* strains *R. tibeticum* CGMCC 1.7071^T^, *R. grahamii* CCGE 502^T^ and *R. mesoamericanum*, all closely related to *R. favelukesii* strains. Cluster II, on scaffold 118.119, contained *exoPOO’MLKHTWV* and an *exoA* pseudogene and shared 99–100% sequence similarity with the LPU83^T^ *exo* cluster harbored on the accessory plasmid LPU83a (NCBI accession number HG916853). In contrast to Cluster I, the *R. favelukesii* Cluster II *exo* genes shared greatest synteny and sequence identity with the *E. meliloti exo* gene cluster, rather than with other *Rhizobium* strains.

All *Medicago* microsymbiont genomes contained the full set of exopolysaccharide biosynthetic genes required to synthesize succinoglycan. The *E. meliloti* strains, including GVPV12 that nodulates *P. vulgaris*, as well as the closely related *Ensifer* sp. 4H41, contained a full set of the required succinoglycan biosynthetic genes, whereas *E. fredii* GR64 and *Ensifer* sp. BR816 did not. Only some *P. vulgaris* microsymbionts, including all those within symbiovar orientale and additionally *R. grahamii* CCGE 502^T^, *R. lusitanum* P1-7^T^, *R. mesoamericanum* strains and *R. freirei* PRF 81^T^, contained the *exoH* gene that is specifically required for the succinylation of succinoglycan (reviewed in Skorupska et al., 2006).

Interestingly, although succinoglycan is required for the effective symbiotic interaction of alfalfa with *E. meliloti*, it does not seem to be necessary for the alfalfa-*R. favelukesii* symbiosis. According to Castellani et al. (2021), LPU83^T^ produces an identical EPS I to the one produced by *E. meliloti* and does not produce EPS II or capsular polysaccharide, yet LPU83^T^ mutants devoid of the genes required for succinoglycan biosynthesis are able to infect and nodulate with alfalfa and are not impaired for symbiosis, compared with the wild type.

#### Characterization of Exopolysaccharide Produced by OR191

**FIGURE 3:**
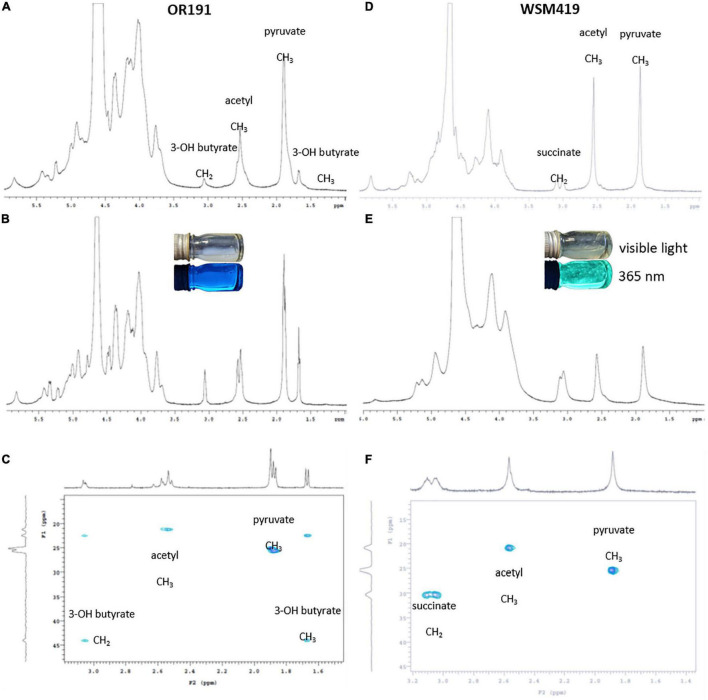
NMR spectra for EPS purified from *Rhizobium favelukesii* OR191 **(A–C)** and *Ensifer medicae* WSM419 **(D,E)**. **(A,D)** ^1^H spectrum at pH 7.0. **(B,E)** ^1^H spectra at pH 5.8. **(C,F)** HSQC-TOCSY. EPS samples containing Calcofuor **(B,E)** were imaged using visible light and at a wavelength of 365 nm.

Following bioinformatics analysis of EPS biosynthetic genes in OR191, we quantified the amount of EPS and substituents produced under neutral and acidic pH growth conditions (Table 4), and determined its composition by NMR spectroscopy. OR191 produced 0.1 mg ml^–1^ and 0.5 mg ml^–1^ EPS at pH 7.0 and 5.8, respectively, representing a fivefold increase in EPS production in acidic conditions. This differs from previous reports of EPS production in *R. favelukesii* LPU83^T^, which found no significant difference in the amount of EPS obtained from cells grown under neutral and acidic conditions (Nilsson et al., 2020). In contrast, the *E. medicae* strain WSM419 produced 0.05 mg ml^–1^ and 4.65 mg ml^–1^ EPS at pH 7.0 and 5.8, respectively, representing a 93-fold increase in EPS production in acidic conditions. This accords with the up-regulation of EPS production at low pH that was previously observed for the succinoglycan-producing strain WSM419 (Dilworth et al., 1999). Purified EPS from WSM419 was highly viscous whereas OR191 EPS was less viscous. In the presence of calcofluor, WSM419 EPS showed an intense greenish fluorescence whereas OR191 EPS gave a distinct blueish fluorescence (Figure 3), indicating structural differences in the EPS.

The ^1^H-NMR pattern for the isolated EPS from OR191 (Figures 3A,B) was consistent with that described by Reeve et al. (1997) for *Rhizobium leguminosarum*. Signals at δ_*H*_ 1.85 and 2.55 were assigned to pyruvyl and acetyl methyl groups, respectively. Absolute values of the chemical shifts are different from those reported in similar EPS (McNeil et al., 1986; Reeve et al., 1997) due to different methods for calibrating the chemical shifts. Signals at δ_*H*_ 3.05 and 1.65 were identified as methylene and methyl, respectively, from a 3-hydroxybutyrate moiety. These assignments were confirmed by correlations between carbons at δ_*C*_ 22 and 24 with the ^1^H signals at 2.05 and 1.65 in the HSQC-TOCSY spectrum (Figure 3C). These correlations confirm that the protons for these two signals must be in the same spin system. The HSQC-TOCSY data also confirmed that assigned pyruvyl and acetyl ^1^H signals were correlated to only a single carbon, consistent with the isolated spin system for each of those methyl groups. The presence of 3-hydroxybutyrate substitution in the OR191 EPS is consistent with the finding that hydroxybutanoyl substituents have been found in the EPS isolated from a variety of *Rhizobium* strains (O’neill et al., 1991).

NMR spectra for the EPS isolated from WMS419 (Figures 3D–F) indicated that this was succinoglycan, with the pattern consistent with that reported previously for this strain (Dilworth et al., 1999) and recently from LPU83^T^ (Castellani et al., 2021). Signals at δ_*H*_ 1.88 and 2.56 were assigned as pyruvyl and acetyl methyls, respectively, and this is supported by correlations of those signals to carbons at δ_*C*_ 25.4 and 20.9 in the HSQC-TOCSY. The signals at δ_*H*_ 3.05 and 3.10 are consistent with those for succinyl methylene as previously reported (Dilworth et al., 1999). An exclusive correlation in the HSQC-TOCSY between a carbon at δ_*C*_ 30.36 and the ^1^H signals at δ_*H*_ 3.05 and 3.10 are consistent with those of a succinic methylene. The broadness of the ^1^H-NMR signals δ_*H*_ 3.0–3.1 in the spectra for the EPS isolated from OR191 and WMS419 suggests that these represent the same structural moiety in the two strains. However, the clear and distinct correlations in the HSQC-TOCSY data clearly indicate that there is no succinate present in OR191 EPS and there is no evidence to indicate the presence of a 3-hydroxybutyl moiety in the EPS isolated from WMS419.

A possible explanation for the difference in EPS produced by OR191 and LPU83^T^ may be that the strains were grown in different media. Osmolarity, ammonium and phosphate availability, and the type of carbon source and age of the culture have been shown to modify the amount and composition of EPS produced by rhizobial strains (Janczarek, 2011). In *E. meliloti*, succinoglycan is essential for establishment of an effective symbiosis with species of *Medicago*, and overexpression of *exoY* in *E. meliloti* 1021, resulting in an increase in succinoglycan production, promoted a more effective symbiosis with *Medicago truncatula* (Jones, 2012). Our results suggest that OR191 does not produce succinoglycan under the given experimental conditions. Future work could look at the genetic regulation of EPS production in OR191 in response to different environmental conditions and different hosts. Creating strains that express succinoglycan could determine whether this EPS improves effectiveness of the OR191 symbiosis with *Medicago* spp.

#### The Role of the *bacA* Gene

*Medicago* hosts belong to a group of legumes within the IRLC that target NCR peptides to their microsymbionts, resulting in endoreduplication, pleomorphism, and terminal differentiation of the bacteroids (reviewed in Alunni and Gourion, 2016). Both the number of NCR peptides and the bacteroid morphotype vary according to the IRLC legume species (Montiel et al., 2017). In contrast to IRLC legumes, phaseoloid legumes such as *P. vulgaris* do not produce NCR peptides, and their bacteroids are not endoreduplicated and remain viable (Mergaert et al., 2006; Haag et al., 2013). The survival of rhizobia within nodules that produce NCR peptides depends on the presence of the rhizobial transporter protein BacA (Alunni and Gourion, 2016). Moreover, the different bacterial orthologs of *bacA* are not functionally interchangeable and the requirement for a specific type of BacA varies according to the particular legume species (Dicenzo et al., 2017). We identified orthologs of *bacA* in all microsymbiont genomes except the two *Paraburkholderia* strains. The OR191 BacA grouped with the *E. meliloti* -type BacA clade (Dicenzo et al., 2017), along with BacA of *R. favelukesii* LPU83^T^, *R. tibeticum* CGMCC 1.7071^T^, *R. grahamii* CCGE 502^T^, *R. mesoamericanum* STM6155 and STM3625, *R. lusitanum* P1-7^T^ and all *E. meliloti* strains. This accords with results obtained by Dicenzo et al. (2017), who found that *E. medicae* and *E. meliloti* BacA had evolved to accommodate a specific interaction with *Medicago* and were divergent from BacA of other rhizobia. Although the *R. favelukesii* BacA is similar to the *E. meliloti* BacA, bacteroids of OR191 and LPU83^T^ show a lack of differentiation inside the nodules (Eardly et al., 1992; Wegener et al., 2001), which has been suggested as an explanation for the low rate of N_2_ fixation in the alfalfa-*R. favelukesii* symbiosis (Eardly et al., 1992; Wegener et al., 2001; Castellani et al., 2021). This also suggests that while the *R. favelukesii* BacA is able to maintain the viability of bacteria inside alfalfa nodule cells, there are differences in the effects of alfalfa NCR peptides on *E. meliloti* bacteroids compared with *R. favelukesii* bacteroids.

## Conclusion

The cultivation of *M. sativa* outside its natural range, in moderately acid agricultural soils, has resulted in this host being nodulated by strains such as *R. favelukesii* OR191, LPU83^T^ and T1155 that are well adapted to these edaphic conditions (Eardly et al., 1985; Del Papa et al., 1999; Bromfield et al., 2010). These *R. favelukesii* strains represent a divergent lineage of acid-adapted strains within the genus *Rhizobium* that are partially effective for N_2_ fixation with both alfalfa and common bean, two hosts that are usually nodulated by distinctly different rhizobial genera.

We identified orthologs of rhizobial acid-adaptation genes in OR191, including *olsC*, which is involved in the production of hydroxylated ornithine lipid species in the acid-tolerant strain *R. tropici* CIAT899^T^ (Rojas-Jiménez et al., 2005; Vences-Guzmán et al., 2011). This gene is absent from the comparatively acid-sensitive *E. medicae* WSM419 and *E. meliloti* 1021 strains. Additionally, *R. favelukesii* contains the highly acid-induced *lpiA* and *acvB* genes, but lacks the associated regulatory system described for this operon in *Ensifer* (Reeve et al., 2006), indicating that an unidentified pH responsive regulatory system is present in *R. favelukesii*. Several mechanisms for acid tolerance in *R. favelukesii* LPU83^T^ have been suggested by Nilsson et al. (2019, 2020).

The *R. favelukesii nod* genes are highly syntenic to the *nod* genes of *E. meliloti Medicago* microsymbionts. The *nodA* gene is closely related to *nodA* of *E. meliloti Medicago* microsymbionts, which is specifically required for the N-acylation of the Nod factor by an unsaturated fatty acid (Debellé et al., 1996). The presence of *nodEF, nodHPQ* and *nodL* suggests that OR191 produces the sulfated, acetylated Nod factors with α, β-unsaturated acyl chains that are required for symbiotic interactions with *Medicago* hosts (Honma et al., 1990; Lerouge et al., 1990; Debellé et al., 2001). However, the Nod factors produced by the closely related strain LPU83^T^ are sulfated and methylated with unsaturated acyl chains (Torres Tejerizo et al., 2011). The *nodG* gene is truncated at the C-terminus in *R. favelukesii* strains, which could decrease *Medicago* nodulation efficiency compared to the *E. meliloti Medicago* microsymbionts. *R. favelukesii* genomes contain additional loci thought to be required for symbiosis with *Medicago*, including genes required for the biosynthesis of succinoglycan. This rhizobial exopolysaccharide is specifically required to prevent the expression of plant defense response genes and allow the formation of infection threads in *Medicago* hosts (Skorupska et al., 2006; Jones and Walker, 2008). We have shown that OR191 does not produce succinoglycan in the conditions tested, which differs from the results found for LPU83^T^ (Castellani et al., 2021); instead, OR191 EPS contains a 3-hydroxybutyrate substituent. The lack of succinoglycan in OR191 may reduce its symbiotic performance with *Medicago* hosts. Additionally, the *R. favelukesii bacA* gene groups with *bacA* genes within the *E. meliloti* clade. *E. meliloti* BacA specifically protects the microsymbiont from the toxic effects of *Medicago* NCR peptides (Dicenzo et al., 2017). These *Medicago*-specific symbiotic determinants in *R. favelukesii* do not prevent *P. vulgaris* nodulation, however, NodL-acetylation or NodS-methylation of the Nod Factor appears to be required for symbiosis with this host. The presence of the nitrogenase genes *nifQWZS* in the genomes of *R. favelukesii* and other *P. vulgaris* microsymbionts, but their absence from the *E. meliloti Medicago* microsymbionts suggests that these genes are important for N_2_ fixation with *P. vulgaris*. Although the *R. favelukesii* strains nodulate and fix with *M. sativa* and *P. vulgaris*, they are poorly effective for N_2_ fixation compared with the usual microsymbionts of these hosts. Future studies will be aimed at identifying genetic determinants required not only for symbiotic interaction with legume hosts but also those that enable effective nitrogen fixation.

## Data Availability Statement

The OR191 genome project is deposited in the Genomes On-Line Database (Reddy et al., 2015) and a high-quality permanent draft genome sequence is deposited in IMG (Markowitz et al., 2014). The data associated with this project is available at https://gold.jgi.doe.gov/project?id=9662 under the project number Gp0009662. Sequencing, finishing, and annotation were performed by the JGI.

## Author Contributions

BE and PB supplied the strain, DNA and background information for this project. BE, WM, JA, JZ, and WR drafted the manuscript. WM, JA, JZ, and WR performed the bioinformatics analyses. DM, MG, PE, RS, TR, NI, AP, TW, and NK involved in sequencing the genome and/or editing the final manuscript. WR and JA extracted EPS from cell cultures. ML and DL performed the NMR spectra analysis of the EPS. All authors read and approved the final manuscript.

## Conflict of Interest

The authors declare that the research was conducted in the absence of any commercial or financial relationships that could be construed as a potential conflict of interest.

## Publisher’s Note

All claims expressed in this article are solely those of the authors and do not necessarily represent those of their affiliated organizations, or those of the publisher, the editors and the reviewers. Any product that may be evaluated in this article, or claim that may be made by its manufacturer, is not guaranteed or endorsed by the publisher.

## Supplementary Material

Supplementary Figure 3 Resident prophages present in *Rhizobium favelukesii* OR191 imaged using PHASTER (Arndt et al., 2016). Scaffolds 4.5 and 84.85, as predicted by PHASTER, contain two putative prophages **(A,C)**, whereas scaffolds 7.8 and 91.92 contain two questionable prophages **(B,D)**, all of which are incomplete.

## Abbreviations

EPS: Exopolysaccharide
FeMo-co: Nitrogenase iron–molybdenum cofactor
GEBA-RNB: Genomic Encyclopedia for Bacteria and Archaea-Root Nodule Bacteria
gANI: Genome Average Nucleotide Identity
GOLD: Genomes Online Database
IMG: Integrated Microbial Genomes
IRLC: Inverted Repeat Lacking Clade
JGI: Joint Genome Institute
NCR peptide: Nodule-specific Cysteine-Rich peptide
TYC: Tryptone yeast extract agar
YEM: Yeast extract mannitol.
