# Supplementary material for: The Genome of the Acid Soil-Adapted Strain Rhizobium favelukesii OR191 Encodes Determinants for Effective Symbiotic Interaction With Both an Inverted Repeat Lacking Clade and a Phaseoloid Legume Host
Source: Front Microbiol. 2022 Apr 13;13:735911. doi: 10.3389/fmicb.2022.735911 (PMC9048898; doi:10.3389/fmicb.2022.735911)
Supplement: Supplementary Figure 3 — Resident prophages present in Rhizobium favelukesii OR191 imaged using PHASTER (Arndt et al., 2016). Scaffolds 4.5 and 84.85, as predicted by PHASTER, contain two putative prophages (A,C), whereas scaffolds 7.8 and 91.92 contain two questionable prophages (B,D), all of which are incomplete. [file Presentation_3.PPTX]

## Slide 1
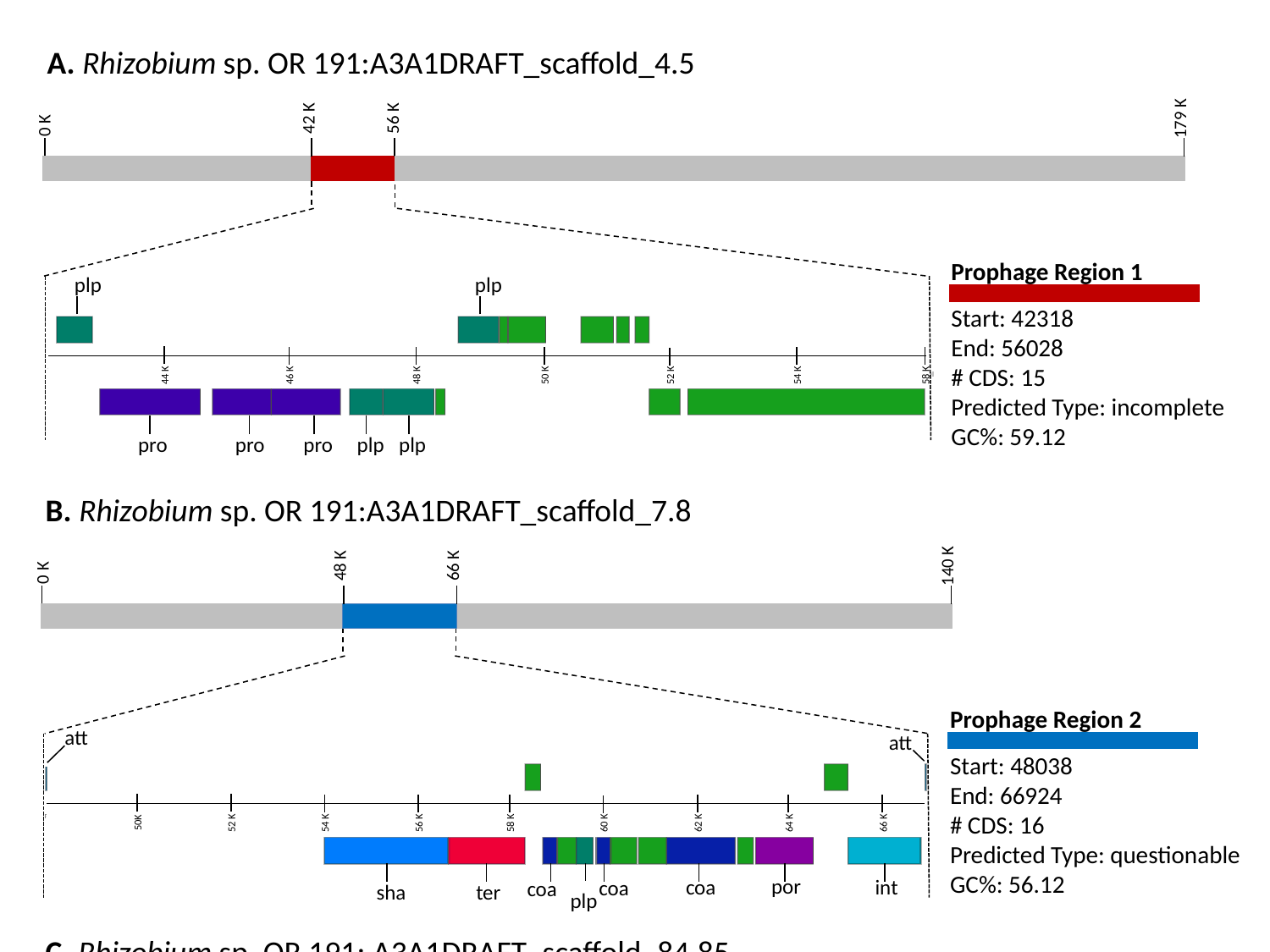

A. Rhizobium sp. OR 191:A3A1DRAFT_scaffold_4.5
56 K
42 K
179 K
0 K
Prophage Region 1
Start: 42318
End: 56028
# CDS: 15
Predicted Type: incomplete
GC%: 59.12
plp plp
44 K
46 K
48 K
50 K
52 K
54 K
58 K
pro pro pro plp plp
B. Rhizobium sp. OR 191:A3A1DRAFT_scaffold_7.8
66 K
48 K
140 K
0 K
Prophage Region 2
Start: 48038
End: 66924
# CDS: 16
Predicted Type: questionable
GC%: 56.12
att
att
50K
52 K
54 K
56 K
58 K
60 K
62 K
64 K
66 K
por
int
coa
coa
coa
sha
ter
plp
C. Rhizobium sp. OR 191: A3A1DRAFT_scaffold_84.85
1.5 K
21 K
20 K
0 K
Prophage Region 3
Start: 1505
End: 20561
# CDS: 29
Predicted Type: incomplete
GC%: 58.95
18 K
10 K
2K
4 K
6 K
8 K
14 K
16 K
20 K
12 K
plp
plp
plp
ter
plp
plp
por
plp
plp
D. Rhizobium sp. OR 191: A3A1DRAFT_scaffold_91.92
18.1 K
0 K
11 K
18 K
Prophage Region 4
Start: 11026
End: 18124
# CDS: 10
Predicted Type: questionable
GC%: 58.12
int
coa
18 K
12 K
13 K
14 K
15 K
16 K
17 K
tra tra plp tra tra
