# Supplementary figures and images for: The Genome of the Acid Soil-Adapted Strain Rhizobium favelukesii OR191 Encodes Determinants for Effective Symbiotic Interaction With Both an Inverted Repeat Lacking Clade and a Phaseoloid Legume Host
Source: Front Microbiol. 2022 Apr 13;13:735911. doi: 10.3389/fmicb.2022.735911 (PMC9048898; doi:10.3389/fmicb.2022.735911)

## Slide 1
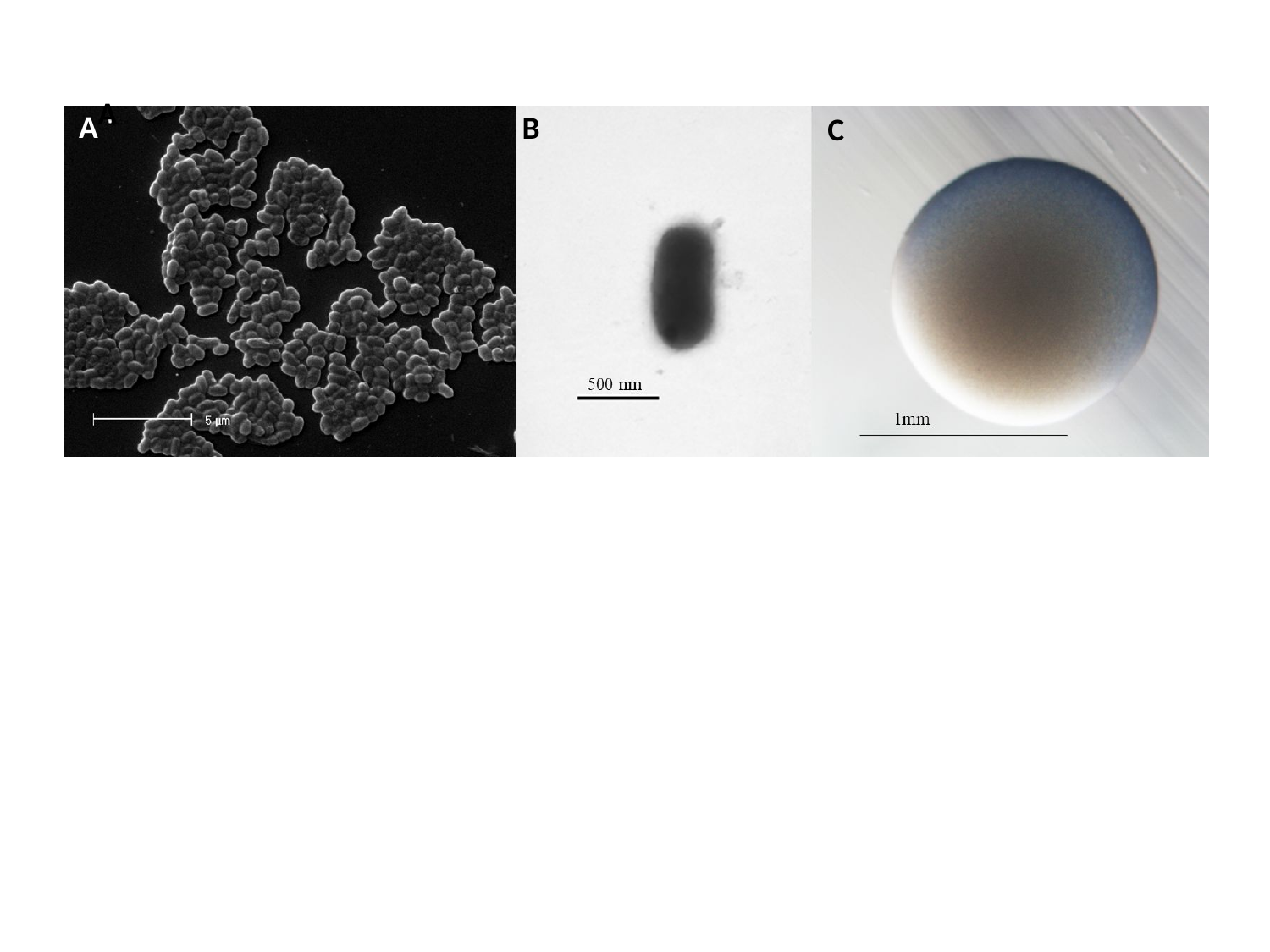

A
A
B
C

Supplement: Supplementary file 2 [file Presentation_1.PPTX]

## Slide 1
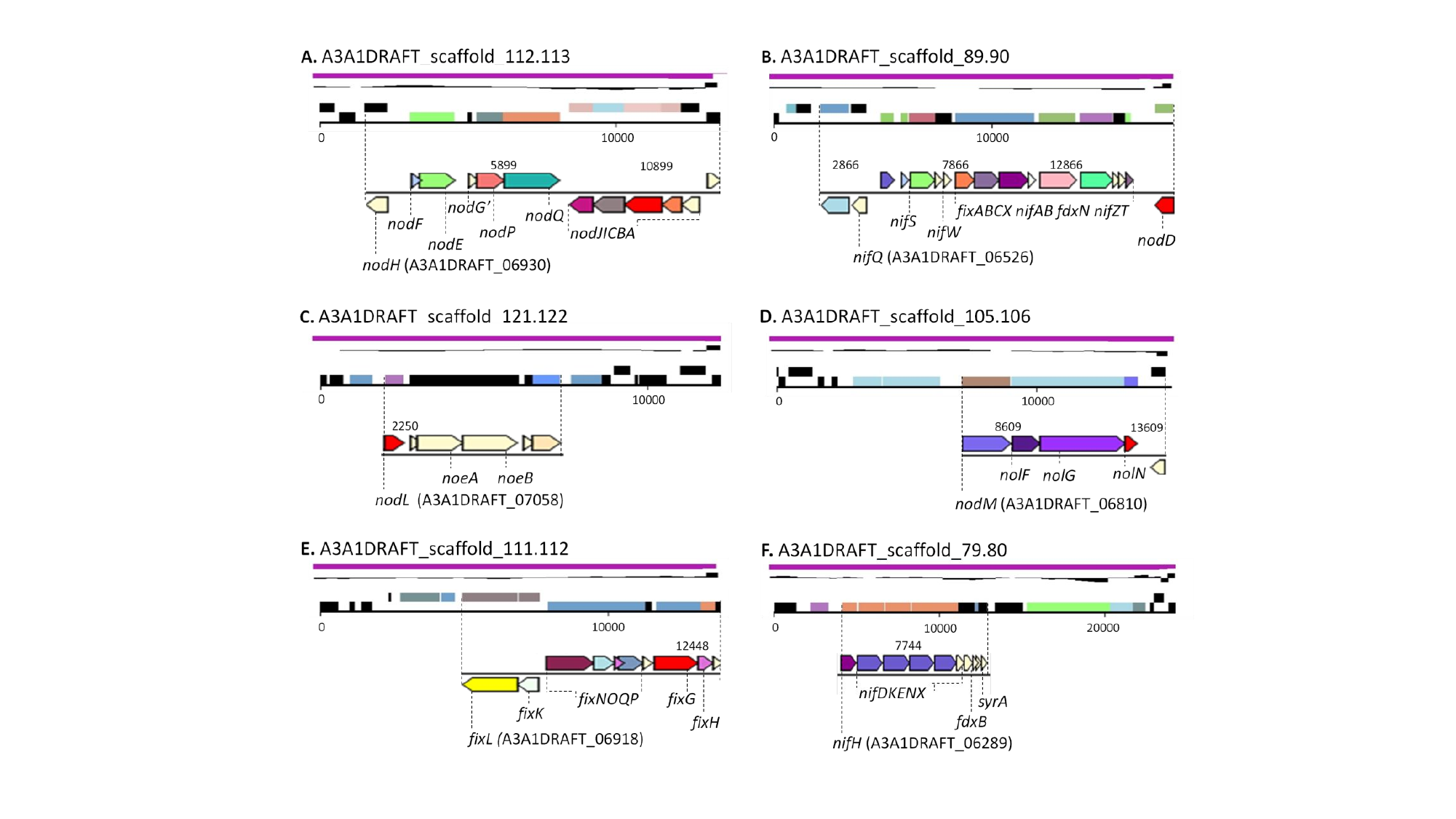

Supplement: Supplementary file 4 [file Presentation_4.PPTX]
