# Supplementary material for: The Genome of the Acid Soil-Adapted Strain Rhizobium favelukesii OR191 Encodes Determinants for Effective Symbiotic Interaction With Both an Inverted Repeat Lacking Clade and a Phaseoloid Legume Host
Source: Front Microbiol. 2022 Apr 13;13:735911. doi: 10.3389/fmicb.2022.735911 (PMC9048898; doi:10.3389/fmicb.2022.735911)
Supplement: Supplementary file 3 [file Presentation_2.PPTX]

## Slide 1
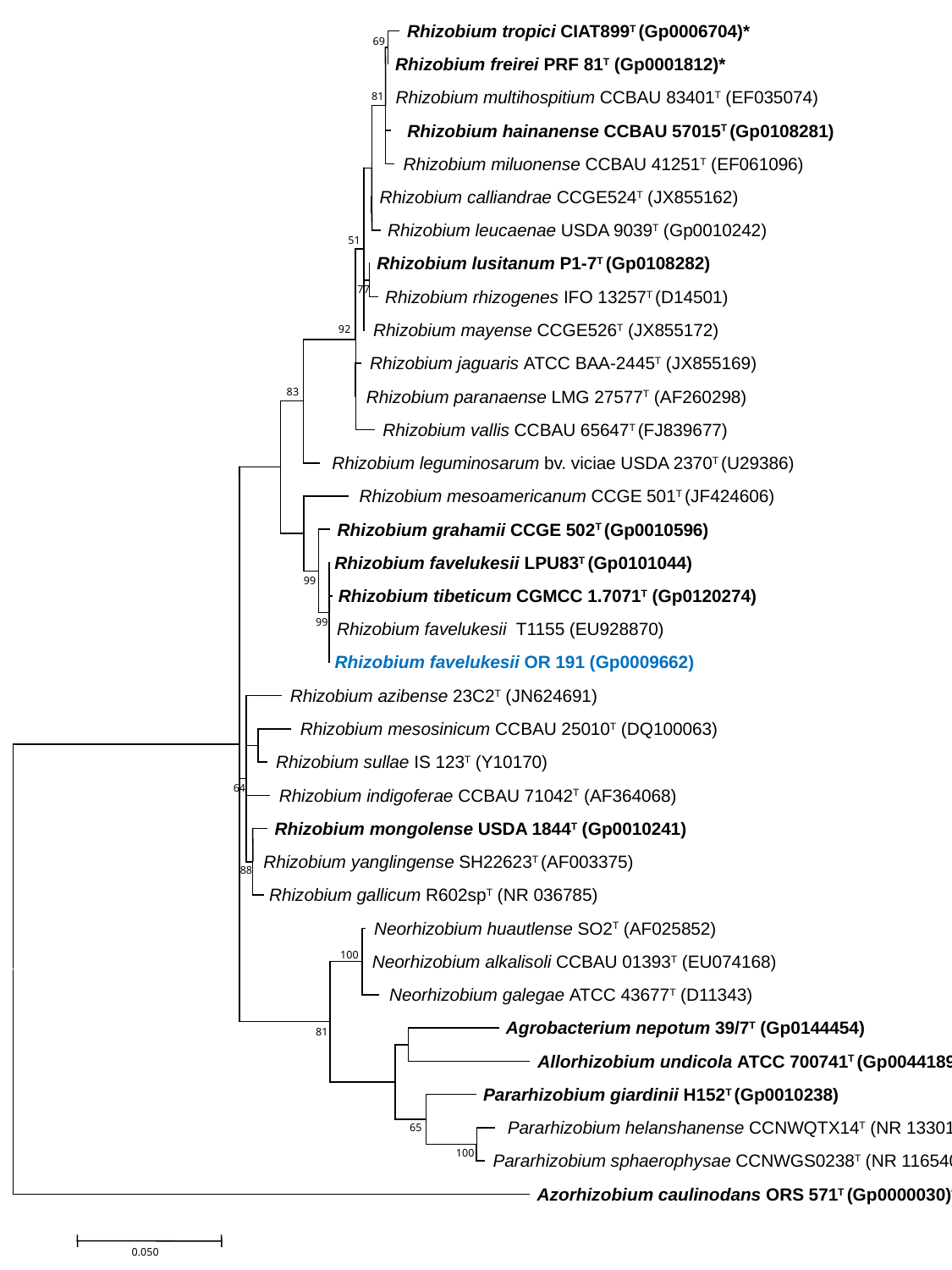

Rhizobium tropici CIAT899T (Gp0006704)*
69
 Rhizobium freirei PRF 81T (Gp0001812)*
 Rhizobium multihospitium CCBAU 83401T (EF035074)
81
 Rhizobium hainanense CCBAU 57015T (Gp0108281)
 Rhizobium miluonense CCBAU 41251T (EF061096)
 Rhizobium calliandrae CCGE524T (JX855162)
 Rhizobium leucaenae USDA 9039T (Gp0010242)
51
 Rhizobium lusitanum P1-7T (Gp0108282)
77
 Rhizobium rhizogenes IFO 13257T (D14501)
 Rhizobium mayense CCGE526T (JX855172)
92
 Rhizobium jaguaris ATCC BAA-2445T (JX855169)
 Rhizobium paranaense LMG 27577T (AF260298)
83
 Rhizobium vallis CCBAU 65647T (FJ839677)
 Rhizobium leguminosarum bv. viciae USDA 2370T (U29386)
 Rhizobium mesoamericanum CCGE 501T (JF424606)
 Rhizobium grahamii CCGE 502T (Gp0010596)
 Rhizobium favelukesii LPU83T (Gp0101044)
99
 Rhizobium tibeticum CGMCC 1.7071T (Gp0120274)
99
 Rhizobium favelukesii T1155 (EU928870)
 Rhizobium favelukesii OR 191 (Gp0009662)
 Rhizobium azibense 23C2T (JN624691)
 Rhizobium mesosinicum CCBAU 25010T (DQ100063)
 Rhizobium sullae IS 123T (Y10170)
64
 Rhizobium indigoferae CCBAU 71042T (AF364068)
 Rhizobium mongolense USDA 1844T (Gp0010241)
 Rhizobium yanglingense SH22623T (AF003375)
88
 Rhizobium gallicum R602spT (NR 036785)
 Neorhizobium huautlense SO2T (AF025852)
100
 Neorhizobium alkalisoli CCBAU 01393T (EU074168)
 Neorhizobium galegae ATCC 43677T (D11343)
 Agrobacterium nepotum 39/7T (Gp0144454)
81
 Allorhizobium undicola ATCC 700741T (Gp0044189 )
 Pararhizobium giardinii H152T (Gp0010238)
 Pararhizobium helanshanense CCNWQTX14T (NR 133019)
65
100
 Pararhizobium sphaerophysae CCNWGS0238T (NR 116540)
 Azorhizobium caulinodans ORS 571T (Gp0000030)*
0.050
