# Supplementary material for: The Genome of the Acid Soil-Adapted Strain Rhizobium favelukesii OR191 Encodes Determinants for Effective Symbiotic Interaction With Both an Inverted Repeat Lacking Clade and a Phaseoloid Legume Host
Source: Front Microbiol. 2022 Apr 13;13:735911. doi: 10.3389/fmicb.2022.735911 (PMC9048898; doi:10.3389/fmicb.2022.735911)
Supplement: Supplementary file 5 [file Table_1.docx]

**Table S1**. Classification, general features and genome sequencing project information for *Rhizobium favelukesii* OR191 in accordance with the MIGS recommendations (Field et al., 2008) published by the Genome Standards Consortium (Field et al., 2011).

| **MIGS ID** | **Property** | **Term** | **Evidence code** | **Reference** |
| --- | --- | --- | --- | --- |
|  | Classification | Domain Bacteria | TAS | Woese et al., 1990; List Editor, 2005 |
|  |  | Phylum *Proteobacteria* | TAS | Garrity et al., 2005b; List Editor, 2005 |
|  |  | Class *Alphaproteobacteria* | TAS | Garrity et al., 2005a; List Editor, 2005 |
|  |  | Order *Rhizobiales* | TAS | Kuykendall, 2005b |
|  |  | Family *Rhizobiaceae* | TAS | Kuykendall, 2005a; List Editor, 2005 |
|  |  | Genus *Rhizobium* | TAS | Kuykendall et al., 2005; List Editor, 2005 |
|  |  | Species *favelukesii* | TAS |  |
|  |  | Strain OR191 |  |  |
|  | Gram stain | Negative | IDA |  |
|  | Cell shape | Rod (0.5 x 1 μm) | IDA |  |
|  | Growth | Fast grower |  |  |
|  | Motility | Motile | IDA |  |
|  | Sporulation | non-sporulating | NAS |  |
|  | Temperature range | Mesophile | NAS |  |
|  | Optimum temperature | 28°C | NAS |  |
|  | pH range, Optimum | ≥5.2; optimum = 7.0 | NAS |  |
| MIGS-1 | INSDC | ATTO00000000 |  |  |
| MIGS-3 | Project name | GEBA-RNB | TAS | Reeve et al., 2015 |
| MIGS-4 | Geographic location | Corvallis, Oregon, USA | TAS | Eardly et al., 1985 |
| MIGS-4.1 | Latitude | 44.63568 | TAS | Eardly et al., 1985 |
| MIGS-4.2 | Longitude | -123.19955 | TAS | Eardly et al., 1985 |
| MIGS-4.3 | Depth | 10 cm | IDA |  |
| MIGS-4.4 | Altitude | 374 m | IDA |  |
| MIGS-5 | Sample collection date | 1982 | TAS | Eardly et al., 1985 |
| MIGS-6 | Habitat | Soil, root nodule, host | TAS | Eardly et al., 1985 |
| MIGS-6.1 | Temperature | 28°C | NAS |  |
| MIGS-6.3 | Salinity | Unknown | NAS |  |
| MIGS 13 | Source Material Identifier | OR191 | TAS | Eardly et al., 1985 |
| MIGS-14 | Pathogenicity | Non-pathogenic | NAS |  |
|  | Biosafety level | 1 | TAS | TRBA 250 |
| MIGS-15 | Biotic relationship | Free living, symbiotic | TAS | Eardly et al., 1985 |
| MIGS-16 | Specific host | *Medicago sativa* | TAS | Eardly et al., 1985 |
| MIGS-17 | Host range | *Medicago sativa* and *Phaseolus vulgaris* | TAS | Eardly et al., 1985 |
| MIGS-22 | Oxygen requirement | Aerobic | IDA |  |
| MIGS-23 | Isolation and growth conditions | YEM, TYC | TAS | Eardly et al., 1985 |
| MIGS 27 | Nucleic acid preparation | CTAB method | TAS | JGI Protocols |
| MIGS-28 | Libraries used | 1x Illumina Std PE library |  |  |
| MIGS-28.1 | Library size | 2,657 Mbp |  |  |
| MIGS-28.2 | Read number | 17,712,488 |  |  |
| MIGS-29 | Sequencing platforms | Illumina HiSeq 2000 |  |  |
| MIGS 30 | Assemblers | Velvet version 1.1.04; Allpaths-LG version r39750 |  |  |
| MIGS-31 | Finishing strategy | High-quality draft |  |  |
| MIGS-31.1 | Contigs | 240 |  |  |
| MIGS-31.2 | Fold coverage | Illumina: 350x |  |  |
| MIGS 32 | Gene calling method | Prodigal 1.4 |  |  |
|  | Locus Tag | A3A1 |  | GOLD |
|  | GenBank ID | ATTO00000000 |  |  |
|  | GenBank Date of Release | July 9, 2013 |  |  |
|  | GOLD ID | Gp0009662 |  | GOLD |
|  | NCBI BIOPROJECT Accession | PRJNA165297 |  | GOLD |

Evidence codes – IDA: Inferred from Direct Assay; TAS: Traceable Author Statement (i.e., a direct report exists in the literature); NAS: Non-traceable Author Statement (i.e., not directly observed for the living, isolated sample, but based on a generally accepted property for the species, or anecdotal evidence). Evidence codes are from the Gene Ontology project (Ashburner et al., 2000; GO Evidence Codes).

**References**

Ashburner, M., Ball, C.A., Blake, J.A., Botstein, D., Butler, H., Cherry, J.M., Davis, A.P., Dolinski, K., Dwight, S.S., Eppig, J.T., Harris, M.A., Hill, D.P., Issel-Tarver, L., Kasarskis, A., Lewis, S., Matese, J.C., Richardson, J.E., Ringwald, M., Rubin, G.M., and Sherlock, G. (2000). Gene ontology: tool for the unification of biology. The Gene Ontology Consortium. *Nature Genetics* 25**,** 25-29.

Eardly, B.D., Hannaway, D.B., and Bottomley, P.J. (1985). Characterization of rhizobia from ineffective alfalfa nodules: ability to nodulate bean plants *Phaseolus vulgaris* (L) Savi]. *Applied and Environmental Microbiology* 50**,** 1422-1427.

Field, D., Amaral-Zettler, L., Cochrane, G., Cole, J.R., Dawyndt, P., Garrity, G.M., Gilbert, J., Glöckner, F.O., Hirschman, L., Karsch-Mizrachi, I., Klenk, H., Knight, R., Kottmann, R., Kyrpides, N.C., Meyer, F., San Gil, I., Sansone, S., Schriml, L.M., Sterk, P., Tatusova, T., Ussery, D.W., White, O., and Wooley, J. (2011). The Genomic Standards Consortium. *PLOS Biology* 9**,** e1001088.

Field, D., Garrity, G., Gray, T., Morrison, N., Selengut, J., Sterk, P., Tatusova, T., Thomson, N., Allen, M., Angiuoli, S.V., Ashburner, M., Axelrod, N., Baldauf, S., Ballard, S., Boore, J.L., Cochrane, G., Cole, J., Dawyndt, P., De Vos, P., De Pamphilis, C., Edwards, R., Faruque, N., Feldman, R., Gilbert, J., Gilna, P., Glöckner, F.O., Goldstein, P., Guralnick, R., Haft, D., Hancock, D., Hermjakob, H., Hertz-Fowler, C., Hugenholtz, P., Joint, I., Kagan, L., Kane, M., Kennedy, J., Kowalchuk, G., Kottmann, R., Kolker, E., Karvitz, S., Kyripides, N., Leebens-Mack, J., Lewis, S., Li, K., Liste, A., Lord, P., Maltsev, N., Markowitz, V., Martiny, J., Methe, B., Moxon, R., Nelson, K., Parkhill, J., Proctor, L., Sansone, S., Spiers, A., Stevens, R., Swift, P., Taylor, C., Tateno, Y., Tett, A., Turner, S., Ussery, D., Vaughan, B., Ward, N., Whetzel, T., Wilson, G., and Wipat, A. (2008). Towards a richer description of our complete collection of genomes and metagenomes "Minimum Information about a Genome Sequence " (MIGS) specification. *Nature Biotechnology* 26**,** 541-547.

Garrity, G.M., Bell, J.A., and Lilburn, T. (2005a). "Class I. Alphaproteobacteria class," in *Bergey's Manual of Systematic Bacteriology,* eds. G.M. Garrity, D.J. Brenner, N.R. Kreig & J.T. Staley. Second ed: New York: Springer - Verlag.

Garrity, G.M., Bell, J.A., and Lilburn, T. (2005b). "Phylum XIV. Proteobacteria phyl. nov.," in *Bergey's Manual of Systematic Bacteriology,* eds. G.M. Garrity, D.J. Brenner, N.R. Kreig & J.T. Staley. Second ed: New York: Springer - Verlag, 1.

GOLD. *Rhizobium favelukesii OR191 project information* [Online]. Available: <https://gold.jgi.doe.gov/projects?id=Gp0009662>

GO Evidence Codes. *Guide to GO Evidence Codes* [Online]. Available: <http://geneontology.org/page/guide-go-evidence-codes>

JGI protocols. *Joint Genome Institute Protocols* [Online]. Available: <http://jgi.doe.gov/user-program-info/pmo-overview/protocols-sample-preparation-information/>

Kuykendall, L.D. (2005a). "Family I. Rhizobiaceae " in *Bergey's Manual of Systematic Bacteriology,* eds. G.M. Garrity, D.J. Brenner, N.R. Krieg & J.T. Staley. New York: Springer - Verlag.

Kuykendall, L.D. (2005b). "Order VI*. Rhizobiales* ord. nov.," in *Bergey's Manual of Systematic Bacteriology,* eds. G.M. Garrity, D.J. Brenner, N.R. Kreig & J.T. Staley. Second ed: New York: Springer - Verlag, 324.

Kuykendall, L.D., Young, J.M., Martínez-Romero, E., Kerr, A., and Sawada, H. (2005). "Genus I. *Rhizobium*," in *Bergey's Manual of Systematic Bacteriology,* eds. G.M. Garrity, D.J. Brenner, N.R. Krieg & J.T. Staley. Second ed (New York: Springer - Verlag).

List Editor. (2005). Validation of publication of new names and new combinations previously effectively published outside the IJSEM. *International Journal of Systematic and Evolutionary Microbiology* 55**,** 2235-2238.

Reeve, W.G., Ardley, J., Tian, R., Eshragi, L., Yoon, J.W., Ngamwisetkun, P., Seshadri, R., Ivanova, N.N., and Kyrpides, N.C. (2015). A genomic encyclopedia of the root nodule bacteria: Assessing genetic diversity through a systematic biogeographic survey. *Standards in Genomic Sciences* 10:14.

TRBA 250. *Biological Agents: Technical Rules for Biological Agents* [Online]. Available: <http://www.baua.de/en/Topics-from-A-to-Z/Biological-Agents/TRBA/TRBA.html>

Woese, C.R., Kandler, O., and Wheelis, M.L. (1990). Towards a natural system of organisms: proposal for the domains Archaea, Bacteria, and Eucarya. *Proceedings of the National Academy of Sciences of the United States of America* 87**,** 4576-4579.
