# Supplementary material for: The Genome of the Acid Soil-Adapted Strain Rhizobium favelukesii OR191 Encodes Determinants for Effective Symbiotic Interaction With Both an Inverted Repeat Lacking Clade and a Phaseoloid Legume Host
Source: Front Microbiol. 2022 Apr 13;13:735911. doi: 10.3389/fmicb.2022.735911 (PMC9048898; doi:10.3389/fmicb.2022.735911)
Supplement: Supplementary file 6 [file Table_2.docx]

**Table S2**. Number of protein coding genes of *Rhizobium favelukesii* OR 191 associated with the general COG functional categories.

| **Code** | **COG Category with extra row at the beginning** | **Gene Count** | **% of total (5,705)** |
| --- | --- | --- | --- |
|  | **CELLULAR PROCESSES AND SIGNALING** |  |  |
| D | Cell cycle control, cell division, chromosome partitioning | 37 | 0.69 |
| M | Cell wall/membrane/envelope biogenesis | 283 | 5.31 |
| N | Cell motility | 75 | 1.41 |
| O | Posttranslational modification, protein turnover, chaperones | 202 | 3.79 |
| T | Signal transduction mechanisms | 230 | 4.32 |
| U | Intracellular trafficking, secretion, and vesicular transport | 64 | 1.20 |
| V | Defense mechanisms | 126 | 2.37 |
| W | Extracellular structures | 16 | 0.30 |
|  | **INFORMATION STORAGE AND PROCESSING** |  |  |
| B | Chromatin structure and dynamics | 1 | 0.02 |
| J | Translation, ribosomal, structure and biogenesis | 228 | 4.28 |
| K | Transcription | 500 | 9.39 |
| L | Replication, recombination and repair | 155 | 2.91 |
|  | **METABOLISM** |  |  |
| C | Energy production and conversion | 330 | 6.19 |
| E | Amino acid transport and metabolism | 538 | 10.1 |
| F | Nucleotide transport and metabolism | 108 | 2.03 |
| G | Carbohydrate transport and metabolism | 521 | 9.78 |
| H | Coenzyme transport and metabolism | 240 | 4.51 |
| I | Lipid transport and metabolism | 228 | 4.28 |
| P | Inorganic ion transport and metabolism | 286 | 5.37 |
| Q | Secondary metabolite biosynthesis, transport and catabolism | 170 | 3.19 |
|  | **POORLY CHARACTERIZED** |  |  |
| R | General function prediction only | 594 | 11.15 |
| S | Function unknown | 339 | 6.36 |
| X | Mobilome: prophages, transposons | 56 | 1.05 |
|  | Not in COGs | 3002 | 38.97 |
