# Supplementary material for: The Genome of the Acid Soil-Adapted Strain Rhizobium favelukesii OR191 Encodes Determinants for Effective Symbiotic Interaction With Both an Inverted Repeat Lacking Clade and a Phaseoloid Legume Host
Source: Front Microbiol. 2022 Apr 13;13:735911. doi: 10.3389/fmicb.2022.735911 (PMC9048898; doi:10.3389/fmicb.2022.735911)
Supplement: Supplementary file 7 [file Table_3.docx]

**Table S3.** Comparison of pH tolerance genes orthologues present in sequenced root nodule bacterial *Rhizobium* strains and in *Ensifer medicae* WSM419 and *Ensifer meliloti* 1021.

| **Strain** | **IMG Genome ID (GOLD ID Gp #)** | | ***kefB*** | | ***gshB*** | | ***cfa*** | | | | | ***pha*** | | | | | | | | ***olsC*** | ***lpiA*** | | | ***acvB*** | | | | ***clc*** | | | | |
| --- | --- | --- | --- | --- | --- | --- | --- | --- | --- | --- | --- | --- | --- | --- | --- | --- | --- | --- | --- | --- | --- | --- | --- | --- | --- | --- | --- | --- | --- | --- | --- | --- |
|  |  |  |  |  |  |  | 1 | 2 | | 3 | | A | | B | C | D | E | F | G |  |  | | |  | | | | *sycA* | | | |  |
| ***Ensifer medicae*** | | | | | | | | | | | | | | | | | | | | | | | | | | | | | | | | |
| WSM419 | 640753051  (Gp0000117) | | 1 | | 1 | | 1 | 1 | | 0 | | 1 | | 1 | 1 | 1 | 1 | 1 | 1 | 0 | 1 | | | 1 | | | | 0 | | | | 0 |
|  |  | |  | |  | |  |  | |  | |  | |  |  |  |  |  |  |  |  | | |  | | | |  | | | |  |
| ***Ensifer meliloti*** | | | | | | | | | | | | | | | | | | | | | | | | | | | | | | | | |
| 1021 | 637190652  Gp0000726 | | 1 | | 1 | | 1 | 1 | | 0 | | 1 | | 1 | 1 | 1 | 1 | 1 | 1 | 0 | 1 | | | 1 | | | | 0 | | | | 0 |
|  |  | |  | |  | |  |  | |  | |  | |  |  |  |  |  |  |  |  | | |  | | | |  | | | |  |
| ***Rhizobium acidisoli*** | | | | | | | | | | | | | | | | | | | | | | | | | | | | | | | | |
| WSM2304 | 643348569  (Gp0001811) | | 1 | | 1 | | 1 | 0 | | 0 | | 1 | | 1 | 1 | 1 | 1 | 1 | 1 | 0 | 1 | | | 1 | | | | 0 | | | | 1 |
| WSM597 | 2509276021  (Gp0007289) | | 1 | | 1 | | 1 | 0 | | 0 | | 1 | | 1 | 1 | 1 | 1 | 1 | 1 | 0 | 2 | | | 1 | | | | 0 | | | | 1 |
|  |  | |  | |  | |  |  | |  | |  | |  |  |  |  |  |  |  |  | | |  | | | |  | | | |  |
| ***Rhizobium etli*** | | | | | | | | | | | | | | | | | | | | | | | | | | | | | | | | |
| CFN 42^T^ | 640427137  (Gp0000395) | | 1 | | 1 | | 1 | 0 | | 0 | | 0 | | 0 | 0 | 0 | 0 | 0 | 0 | 0 | 1 | | | 1 | | | | 0 | | | | 1 |
|  |  | |  | |  | |  |  | |  | |  | |  |  |  |  |  |  |  |  | | |  | | | |  | | | |  |
| ***Rhizobium favelukesii*** | | | | | | | | | | | | | | | | | | | | | | | | | | | | | | | | |
| OR191 | 2513237138  (Gp0009662) | | 1 | | 1 | | 1 | 0 | | 0 | | 1 | | 1 | 1 | 1 | 1 | 1 | 1 | 1 | 1 | | | 1 | | | | 0 | | | | 0 |
| LPU83^T^ | 2597490046  (Gp0101044) | | 1 | | 1 | | 1 | 0 | | 0 | | 1 | | 1 | 1 | 1 | 1 | 1 | 1 | 1 | 1 | | | 1 | | | | 0 | | | | 0 |
|  |  | |  | |  | |  |  | |  | |  | |  |  |  |  |  |  |  |  | | |  | | | |  | | | |  |
| ***Rhizobium freirei*** | | | | | | | | | | | | | | | | | | | | | | | | | | | | | | | | |
| PRF 81^T^ | 2545824643  (Gp0001812) | | 1 | | 1 | | 1 | 1 | | 0 | | 1 | | 1 | 1 | 1 | 1 | 1 | 1 | 1 | 1 | | | 1 | | | | 1 | | | | 1 |
|  |  | |  | |  | |  |  | |  | |  | |  |  |  |  |  |  |  |  | | |  | | | |  | | | |  |
| ***Rhizobium grahamii*** | | | | | | | | | | | | | | | | | | | | | | | | | | | | | | | | |
| CCGE 502^T^ | 2534681796  (Gp0010596) | | 1 | | 1 | | 1 | 0 | | 0 | | 1 | | 1 | 1 | 1 | 1 | 1 | 1 | 1 | 1 | | | 1 | | | 0 | | | | 1 | |
|  |  | |  | |  | |  |  | |  | |  | |  |  |  |  |  |  |  |  |  | | |  | | | |  | | | |
| ***Rhizobium hainanense*** | | | | | | | | | | | | | | | | | | | | | | | | | | | | | | | | |
| CCBAU 57015^T^ | 2615840609  (Gp0108281) | | 1 | | 1 | | 1 | 1 | | 1 | | 1 | | 1 | 1 | 1 | 1 | 1 | 1 | 1 | 1 | 1 | | | 1 | | | | 1 | | | |
|  |  | |  | |  | |  |  | |  | |  | |  |  |  |  |  |  |  |  |  | | |  | | | |  | | | |
| ***Rhizobium hidalgonense*** | | | | | | | | | | | | | | | | | | | | | | | | | | | | | | | | |
| CB782 | 2510065076  (Gp0007437) | | 1 | | 1 | | 1 | 0 | | 0 | | 1 | | 1 | 1 | 1 | 1 | 1 | 1 | 0 | 1 | 1 | | | 0 | | | | 1 | | | |
| WSM2012 | 2509276033  (Gp0007352) | | 1 | | 1 | | 1 | 0 | | 0 | | 1 | | 1 | 1 | 1 | 1 | 1 | 1 | 0 | 1 | 1 | | | 0 | | | | 1 | | | |
|  |  | |  | |  | |  |  | |  | |  | |  |  |  |  |  |  |  |  |  | | |  | | | |  | | | |
| ***Rhizobium leguminosarum* (Genospecies B)** | | | | | | | | | | | | | | | | | | | | | | | | | | | | | | | | |
| 3841 | 639633055  (Gp0000346) | | 1 | | 1 | | 1 | 0 | | 0 | | 1 | | 1 | 1 | 1 | 1 | 1 | 1 | 0 | 1 | 1 | | | 0 | | | | 0 | | | |
| VF39 | 2513237103  (Gp0009626) | | 1 | | 1 | | 1 | 0 | | 0 | | 1 | | 1 | 1 | 1 | 1 | 1 | 1 | 0 | 1 | 1 | | | 0 | | | | 0 | | | |
|  |  | |  | |  | |  |  | |  | |  | |  |  |  |  |  |  |  |  |  | | |  | | | |  | | | |
| ***Rhizobium leguminosarum* (Genospecies C)** | | | | | | | | | | | | | | | | | | | | | | | | | | | | | | | | |
| GB30 | 2513237162  (Gp00097658) | | 1 | | 1 | | 1 | 0 | | 0 | | 1 | | 1 | 1 | 1 | 1 | 1 | 1 | 0 | 1 | 1 | | | 0 | | | | 1 | | | |
| Ps8 | 2515154116  (Gp0009622) | | 1 | | 1 | | 1 | 0 | | 0 | | 1 | | 1 | 1 | 1 | 1 | 1 | 1 | 0 | 1 | 1 | | | 0 | | | | 1 | | | |
| TA1 | 2510461076  (Gp0007391) | | 1 | | 1 | | 1 | 0 | | 0 | | 1 | | 1 | 1 | 1 | 1 | 1 | 1 | 0 | 1 | 1 | | | 0 | | | | 1 | | | |
| Vc2 | 2515154113  (Gp0009623) | | 1 | | 1 | | 1 | 0 | | 0 | | 1 | | 1 | 1 | 1 | 1 | 1 | 1 | 0 | 1 | 1 | | | 0 | | | | 0 | | | |
| Vh3 | 2515154114  (Gp0009624) | | 1 | | 1 | | 1 | 0 | | 0 | | 1 | | 1 | 1 | 1 | 1 | 1 | 1 | 0 | 1 | 1 | | | 0 | | | | 0 | | | |
|  |  | |  | |  | |  |  | |  | |  | |  |  |  |  |  |  |  |  |  | | |  | | | |  | | | |
| ***Rhizobium leguminosarum* s.s. (Genospecies E)** | | | | | | | | | | | | | | | | | | | | | | | | | | | | | | | | |
| 4292 | 2516653085  (Gp0010236) | | 1 | | 1 | | 1 | 0 | | 0 | | 1 | | 1 | 1 | 1 | 1 | 1 | 1 | 0 | 1 | 1 | | | 0 | | | | 1 | | | |
| 128C53 | 2515154115  (Gp0009628) | | 1 | | 1 | | 1 | 0 | | 0 | | 1 | | 1 | 1 | 1 | 1 | 1 | 1 | 0 | 1 | 1 | | | 0 | | | | 1 | | | |
| CC283b | 2507525018  (Gp0007356) | | 1 | | 1 | | 1 | 0 | | 0 | | 1 | | 1 | 1 | 1 | 1 | 1 | 1 | 0 | 1 | 1 | | | 0 | | | | 1 | | | |
| UPM1137 | 2513237085  (Gp0009758) | | 1 | | 1 | | 1 | 0 | | 0 | | 1 | | 1 | 1 | 1 | 1 | 1 | 1 | 0 | 1 | 1 | | | 0 | | | | 0 | | | |
|  |  | |  | |  | |  |  | |  | |  | |  |  |  |  |  |  |  |  |  | | |  | | | |  | | | |
| ***Rhizobium leguminosarum* (Genospecies H)** | | | | | | | | | | | | | | | | | | | | | | | | | | | | | | | | |
| SRDI943 | 2517093000  (Gp0009787) | | 1 | | 1 | | 1 | 0 | | 0 | | 1 | | 1 | 1 | 1 | 1 | 1 | 1 | 0 | 1 | 1 | | | 0 | | | | 1 | | | |
| WSM1325 | 644736401  (Gp0001809) | | 1 | | 1 | | 1 | 0 | | 0 | | 1 | | 1 | 1 | 1 | 1 | 1 | 1 | 0 | 1 | 1 | | | 0 | | | | 1 | | | |
|  |  | |  | |  | |  |  | |  | |  | |  |  |  |  |  |  |  |  |  | | |  | | | |  | | | |
| ***Rhizobium leguminosarum* Genospecies J** | | | | | | | | | | | | | | | | | | | | | | | | | | | | | | | | |
| WSM1455 | 2509276044  (Gp0007354) | | 1 | | 1 | | 1 | 0 | | 0 | | 1 | | 1 | 1 | 1 | 1 | 1 | 1 | 0 | 1 | 1 | | | 1 | | | | 0 | | | |
| WSM1481 | 2516653077  (Gp0009625) | | 1 | | 1 | | 1 | 0 | | 0 | | 1 | | 1 | 1 | 1 | 1 | 1 | 1 | 0 | 1 | 1 | | | 1 | | | | 0 | | | |
|  |  | |  | |  | |  |  | |  | |  | |  |  |  |  |  |  |  |  |  | | |  | | | |  | | | |
| ***Rhizobium leguminosarum* Genospecies K** | | | | | | | | | | | | | | | | | | | | | | | | | | | | | | | | |
| FA23 | 2513237093  (Gp0009659) | | 1 | | 1 | | 1 | 0 | | 0 | | 1 | | 1 | 1 | 1 | 1 | 1 | 1 | 0 | 1 | 1 | | | 0 | | | | 0 | | | |
|  |  | |  | |  | |  |  | |  | |  | |  |  |  |  |  |  |  |  |  | | |  | | | |  | | | |
| ***Rhizobium leguminosarum* Genospecies M** | | | | | | | | | | | | | | | | | | | | | | | | | | | | | | | | |
| SRDI565 | 2517287029  (Gp0009788) | | 1 | | 1 | | 1 | 0 | | 0 | | 1 | | 1 | 1 | 1 | 1 | 1 | 1 | 0 | 1 | 1 | | | 0 | | | | 1 | | | |
|  |  | |  | |  | |  |  | |  | |  | |  |  |  |  |  |  |  |  |  | | |  | | | |  | | | |
| ***Rhizobium leguminosarum* Genospecies N** | | | | | | | | | | | | | | | | | | | | | | | | | | | | | | | | |
| TOM | 2516653047  (Gp0009627) | | 1 | | 1 | | 1 | 0 | | 0 | | 1 | | 1 | 1 | 1 | 1 | 1 | 1 | 0 | 1 | 1 | | | 0 | | | | 0 | | | |
|  |  | |  | |  | |  |  | |  | |  | |  |  |  |  |  |  |  |  |  | | |  | | | |  | | | |
| ***Rhizobium leguminosarum* Genospecies O** | | | | | | | | | | | | | | | | | | | | | | | | | | | | | | | | |
| UPM1131 | 2513237084  (Gp0009782) | | 1 | | 1 | | 1 | 0 | | 0 | | 1 | | 1 | 1 | 1 | 1 | 1 | 1 | 0 | 1 | 1 | | | 0 | | | | 0 | | | |
|  |  | |  | |  | |  |  | |  | |  | |  |  |  |  |  |  |  |  |  | | |  | | | |  | | | |
| ***Rhizobium leguminosarum* Genospecies Q** | | | | | | | | | | | | | | | | | | | | | | | | | | | | | | | | |
| 248 | 2515075009  (Gp0010237) | | 1 | | 1 | | 1 | 0 | | 0 | | 1 | | 1 | 1 | 1 | 1 | 1 | 1 | 0 | 1 | 1 | | | 0 | | | | 0 | | | |
|  |  | |  | |  | |  |  | |  | |  | |  |  |  |  |  |  |  |  |  | | |  | | | |  | | | |
| ***Rhizobium leguminosarum s.l.*** | | | | | | | | | | | | | | | | | | | | | | | | | | | | | | | | |
| CC278f | 2509276052  (Gp0007351) | | 1 | | 1 | | 1 | 0 | | 0 | | 1 | | 1 | 1 | 1 | 1 | 1 | 1 | 0 | 1 | 1 | | | 0 | | | | 0 | | | |
| WSM1689 | 2510065019  (Gp0007438) | | 1 | | 1 | | 1 | 0 | | 0 | | 1 | | 1 | 1 | 1 | 1 | 1 | 1 | 0 | 1 | 1 | | | 0 | | | | 0 | | | |
|  |  | |  | |  | |  |  | |  | |  | |  |  |  |  |  |  |  |  |  | | |  | | | |  | | | |
| ***Rhizobium leucaenae*** | | | | | | | | | | | | | | | | | | | | | | | | | | | | | | | | |
| USDA 9039^T^ | 2524023209  (Gp0010242) | | 1 | | 1 | | 1 | 1 | | 0 | | 1 | | 1 | 1 | 1 | 1 | 1 | 1 | 1 | 1 | 1 | | | 0 | | | | 1 | | | |
|  |  | |  | |  | |  |  | |  | |  | |  |  |  |  |  |  |  |  |  | | |  | | | |  | | | |
| ***Rhizobium lusitanum*** | | | | | | | | | | | | | | | | | | | | | | | | | | | | | | | | |
| P1-7^T^ | 2615840626  (Gp0108282) | | 1 | | 1 | | 1 | 1 | | 0 | | 1 | | 1 | 1 | 1 | 1 | 1 | 1 | 1 | 2 | 2 | | | 1 | | | | 2 | | | |
|  |  | |  | |  | |  |  | |  | |  | |  |  |  |  |  |  |  |  |  | | |  | | | |  | | | |
| ***Rhizobium mesoamericanum*** | | | | | | | | | | | | | | | | | | | | | | | | | | | | | | | | |
| STM3625 | 2534682333  (Gp0023271) | | 1 | | 1 | | 1 | 1 | | 0 | | 1 | | 1 | 1 | 1 | 1 | 1 | 1 | 1 | 1 | 1 | | | 0 | | | | 0 | | | |
| STM6155 | 2513237088  (Gp0009783) | | 1 | | 1 | | 1 | 0 | | 0 | | 1 | | 1 | 1 | 1 | 1 | 1 | 1 | 1 | 1 | 1 | | | 0 | | | | 0 | | | |
|  |  | |  | |  | |  |  | |  | |  | |  |  |  |  |  |  |  |  |  | | |  | | | |  | | | |
| ***Rhizobium miluonense*** | | | | | | | | | | | | | | | | | | | | | | | | | | | | | | | | |
| HAMBI 2971^T^ | 2617270742  (Gp0108283) | | 1 | | 1 | | 1 | 1 | | 0 | | 1 | | 1 | 1 | 1 | 1 | 1 | 1 | 1 | 1 | 1 | | | 1 | | | | 1 | | | |
|  |  | |  | |  | |  |  | |  | |  | |  |  |  |  |  |  |  |  |  | | |  | | | |  | | | |
| ***Rhizobium mongolense*** | | | | | | | | | | | | | | | | | | | | | | | | | | | | | | | | |
| USDA 1844^T^ | 2513237146  (Gp0010241) | | 1 | | 1 | | 1 | 0 | | 0 | | 1 | | 1 | 1 | 1 | 1 | 1 | 1 | 0 | 1 | 1 | | | 0 | | | | 0 | | | |
|  |  | |  | |  | |  |  | |  | |  | |  |  |  |  |  |  |  |  |  | | |  | | | |  | | | |
| ***Rhizobium multihospitium*** | | | | | | | | | | | | | | | | | | | | | | | | | | | | | | | | |
| HAMBI 2975^T^ | 2615840698  (Gp0108284) | | 1 | | 1 | | 1 | 1 | | 1 | | 1 | | 1 | 1 | 1 | 1 | 1 | 1 | 1 | 1 | 1 | | | 1 | | | | 1 | | | |
|  |  | |  | |  | |  |  | |  | |  | |  |  |  |  |  |  |  |  |  | | |  | | | |  | | | |
| ***Rhizobium phaseoli*** | | | | | | | | | | | | | | | | | | | | | | | | | | | | | | | | |
| CIAT 652 | 642555152  (Gp0003237) | 1 | | 1 | | 1 | | | 0 | 0 | | 1 | | 1 | 1 | 1 | 1 | 1 | 1 | 0 | 1 | | 1 | | | 0 | | | | 0 | | |
|  |  |  | |  | |  | | |  |  |  | |  | |  |  |  |  |  |  |  | |  | | |  | | | |  | | |
| ***Rhizobium* sp.** | | | | | | | | | | | | | | | | | | | | | | | | | | | | | | | | |
| WSM2297 | 2508501100  (Gp0007348) | 1 | | 1 | | 1 | | | 0 | 0 | 1 | | 1 | | 1 | 1 | 1 | 1 | 1 | 0 | 1 | | 1 | | | 0 | | | | 1 | | |
|  |  |  | |  | |  | | |  |  |  | |  | |  |  |  |  |  |  |  | |  | | |  | | | |  | | |
| ***Rhizobium sullae*** | | | | | | | | | | | | | | | | | | | | | | | | | | | | | | | | |
| WSM1592 | 2513237144  (Gp0010240) | | 1 | | 1 | | 1 | 0 | | 0 | | 1 | | 1 | 1 | 1 | 1 | 1 | 1 | 0 | 1 | 1 | | | 0 | | | | 0 | | | |
|  |  | |  | |  | |  |  | |  | |  | |  |  |  |  |  |  |  |  |  | | |  | | | |  | | | |
| ***Rhizobium tibeticum*** | | | | | | | | | | | | | | | | | | | | | | | | | | | | | | | | |
| CGMCC 1.7071^T^ | 2671180023  (Gp0120274) | | 1 | | 1 | | 1 | 0 | | 0 | | 1 | | 1 | 1 | 1 | 1 | 1 | 1 | 1 | 1 | 1 | | | 0 | | | | 1 | | | |
|  |  | |  | |  | |  |  | |  | |  | |  |  |  |  |  |  |  |  |  | | |  | | | |  | | | |
| ***Rhizobium tropici*** | | | | | | | | | | | | | | | | | | | | | | | | | | | | | | | | |
| CIAT899^T^ | 2524023199  (Gp0006704) | | 1 | | 1 | | 1 | 1 | | 1 | | 1 | | 1 | 1 | 1 | 1 | 1 | 1 | 1 | 1 | 1 | | | 1 | | | | 1 | | | |
