# Supplementary material for: The Genome of the Acid Soil-Adapted Strain Rhizobium favelukesii OR191 Encodes Determinants for Effective Symbiotic Interaction With Both an Inverted Repeat Lacking Clade and a Phaseoloid Legume Host
Source: Front Microbiol. 2022 Apr 13;13:735911. doi: 10.3389/fmicb.2022.735911 (PMC9048898; doi:10.3389/fmicb.2022.735911)
Supplement: Supplementary file 8 [file Table_4.docx]

**Table S4**. List of the 39 sequenced rhizobial strains used in comparative genome analyses of *Medicago* and *Phaseolus vulgaris* microsymbionts

| **Strain** | | **IMG Genome ID (GOLD ID Gp #)** | | **Genome size (bp)** | | **Primary host** | **Nod+ Fix+ on *Phaseolus vulgaris*** | | **Geographic origin** | | **NodA Group^a^** | | **Reference** | |  |
| --- | --- | --- | --- | --- | --- | --- | --- | --- | --- | --- | --- | --- | --- | --- | --- |
| ***Ensifer fredii*** | |  | |  | |  |  | |  | |  | |  | |  |
| GR64 | | 2547132261 (Gp0008742) | | 6959392 | | *Phaseolus vulgaris* | Y | | Spain | | F | | (Torres Tejerizo et al., 2012) | |  |
| ***Ensifer meliloti*** | |  | |  | |  |  | |  | |  | |  | |  |
| 1021 | | 637000269 (Gp0000726) | | 6691694 | | *Medicago sativa* |  | | Australia | | A | | (Galibert et al., 2001) | |  |
| 2011 | | 2562617130 (Gp0037090) | | 6693185 | | *Medicago sativa* |  | | Australia | | A | | (Sallet et al., 2013) | |  |
| 5A14 | | 2551306084  (Gp0021882) | | 8942552 | | *Medicago sativa* |  | | Iran | | A | | (Galardini et al., 2013b) | |  |
| AE608H | | 2551306085  (Gp0021893) | | 7347181 | | *Medicago sativa* |  | | Italy | | A | | (Galardini et al., 2013b) | |  |
| AK58 | | 2511231052  (Gp0007938) | | 6974333 | | *Medicago falcata* |  | | Kazakhstan | | A | | (Galardini et al., 2013a) | |  |
| AK83 | | 650716086  (Gp0006695) | | 7140471 | | *Medicago falcata* |  | | Kazakhstan | | A | | (Galardini et al., 2011) | |  |
| BL225C | | 648276728  (Gp0006560) | | 6968865 | | *Medicago sativa* |  | | Italy | | A | | (Galardini et al., 2011) | |  |
| BO21CC | | 2516653046  (Gp0008290) | | 6989037 | | *Medicago sativa* |  | | Italy | | A | | (Galardini et al., 2013a) | |  |
| CCNWSX0020 | | 2519103084  (Gp0016493) | | 7001588 | | *Medicago lupulina* |  | | China | | A | | (Li et al., 2012) | |  |
| CIAM1775 | | 2510065056  (Gp0009789) | | 6712855 | | *Medicago lupulina* |  | | Kazakhstan | | A | | (Reeve et al., 2015) | |  |
| GR4 | | 2523533632  (Gp0020501) | | 7139558 | | *Medicago sativa* |  | | Spain | | A | | (Martínez-Abarca et al., 2013) | |  |
| GVPV12 | | 2513237140  (Gp0010269) | | 7076289 | | *Phaseolus vulgaris* | Y | | Spain | | F | | (Reeve et al., 2015) | |  |
| Mlalz-1 | | 2513237143  (Gp0010229) | | 6664116 | | *Medicago laciniata* |  | | Spain | | A | | (Osman et al., 2017) | |  |
| MVII-I | | 2513237086  (Gp0010230) | | 7265287 | | *Medicago sativa* |  | | Germany | | A | | (Reeve et al., 2015) | |  |
| Rm41 | | 2561511187  (Gp0025853) | | 7149690 | | *Medicago sativa* |  | | Hungary | | A | | (Weidner et al., 2013) | |  |
| RRl128 | | 2513237091  (Gp0010231) | | 6900273 | | *Medicago truncatula* |  | | Australia | | A | | (Reeve et al., 2014) | |  |
| SM11 | 651053067  (Gp0006018) | | 7173736 | | *Medicago sativa* | | |  | | Germany | | A | | (Schneiker-Bekel et al., 2011) | |
| WSM1022 | 2510065057  (Gp0010232) | | 6649661 | | *Medicago orbicularis* | | |  | | Greece | | A | | (Terpolilli et al., 2013) | |
| ***Ensifer* sp.** |  | |  | |  | | |  | |  | |  | |  | |
| 4H41 | 2515154107 (Gp0010268) | | 6795637 | | *Phaseolus vulgaris* | | | Y | | Tunisia | | F | | [47] | |
| BR816 | 2516143018  (Gp0010267) | | 6951533 | | *Leucaena leucocephala* | | | Y | | Brazil | | F | | (Reeve et al., 2015) | |
| ***Paraburkholderia dilworthii*** |  | |  | |  | | |  | |  | |  | |  | |
| WSM3556^T^ | 2508501124 (Gp0010131) | | 7679067 | | *Lebeckia ambigua* | | | Y | | South Africa | | D | | (De Meyer et al., 2015a) | |
| ***Paraburkholderia tuberum*** |  | |  | |  | | |  | |  | |  | |  | |
| WSM4176 | 2516653074 (Gp0010127) | | 9065247 | | *Lebeckia ambigua* | | | Y | | South Africa | | D | | (De Meyer et al., 2015b) | |
| ***Rhizobium aethiopicum*** |  | |  | |  | | |  | |  | |  | |  | |
| HBR26^T^ | 2615840624  (Gp0108286) | | 6557588 | | *Phaseolus vulgaris* | | | Y | | Ethiopia | | C | | (Aserse et al., 2017) | |
| ***Rhizobium etli*** |  | |  | |  | | |  | |  | |  | |  | |
| CFN 42^T^ | 640427137  (Gp0000395) | | 6530228 | | *Phaseolus vulgaris* | | | Y | | Mexico | | C | | (González et al., 2006) | |
| ***Rhizobium favelukesii*** |  | |  | |  | | |  | |  | |  | |  | |
| LPU83^T^ | 2740891995  (Gp0101044) | | 7569648 | | *Medicago sativa* | | | Y | | Argentina | | B | | (Wibberg et al., 2014) | |
| OR191 | 2513237138  (Gp0009662) | | 7368160 | | *Medicago sativa* | | | Y | | USA | | B | | (Reeve et al., 2015) | |
| ***Rhizobium freirei*** |  | |  | |  | | |  | |  | |  | |  | |
| PRF 81^T^ | 2545824643 (Gp0001812) | | 7083871 | | *Phaseolus vulgaris* | | | Y | | Brazil | | G | | (Ormeño-Orrillo et al., 2012) | |
| ***Rhizobium grahamii*** |  | |  | |  | | |  | |  | |  | |  | |
| CCGE 502^T^ | 2534681796  (Gp0010596) | | 7146037 | | *Dalea leporina* | | | Y | | Mexico | | F | | (Althabegoiti et al., 2012) | |
| ***Rhizobium leguminosarum s.s.*** |  | |  | |  | | |  | |  | |  | |  | |
| 4292 | 2516653085  (Gp0010236) | | 7346596 | | *Phaseolus vulgaris* | | | Y | | UK | | C | | (Reeve et al., 2015) | |
| ***Rhizobium leguminosarum* Genospecies K** |  | |  | |  | | |  | |  | |  | |  | |
| FA23 | 2513237093  (Gp0009659) | | 7545552 | | *Phaseolus vulgaris* | | | Y | | Poland | | C | | (Reeve et al., 2015) | |
| ***Rhizobium leucaenae*** |  | |  | |  | | |  | |  | |  | |  | |
| USDA 9039^T^ | 2524023209  (Gp0010242) | | 6679728 | | *Phaseolus vulgaris* | | | Y | | Brazil | | G | | (Reeve et al., 2015) | |
| ***Rhizobium lusitanum*** |  | |  | |  | | |  | |  | |  | |  | |
| P1-7^T^ | 2615840626  (Gp0108282) | | 7921970 | | *Phaseolus vulgaris* | | | Y | | Portugal | | G | | (Valverde et al., 2006;Whitman et al., 2015) | |
| ***Rhizobium mesoamericanum*** |  | |  | |  | | |  | |  | |  | |  | |
| STM3625 | 2534682333  (Gp0023271) | | 6453427 | | *Mimosa pudica* | | | Y | | French Guiana | | E | | (Moulin et al., 2013) | |
| STM6155 | 2513237088  (Gp0009783) | | 6927906 | | *Mimosa pudica* | | | Y | | New Caledonia | | E | | (Klonowska et al., 2017) | |
| ***Rhizobium mongolense*** |  | |  | |  | | |  | |  | |  | |  | |
| USDA 1844^T^ | 2599185170  (Gp0104823) | | 7295545 | | *Medicago ruthenica* | | | Y | | Mongolia | | B | | (Reeve et al., 2015) | |
| ***Rhizobium phaseoli*** |  | |  | |  | | |  | |  | |  | |  | |
| CIAT 652 | 642555152  (Gp0003237) | | 6448048 | | *Phaseolus vulgaris* | | | Y | | Colombia | | C | | (López-Guerrero et al., 2012) | |
| ***Rhizobium tibeticum*** |  | |  | |  | | |  | |  | |  | |  | |
| CGMCC 1.7071^T^ | 2671180023  (Gp0120274) | | 7065782 | | *Trigonella archiducis-nicolai* | | | Y | | China (Tibet) | | B | | (Hou et al., 2009;Torres Tejerizo et al., 2017) | |
| ***Rhizobium tropici*** |  | |  | |  | | |  | |  | |  | |  | |
| CIAT899^T^ | 2524023199  (Gp0006704) | | 6686334 | | *Phaseolus vulgaris* | | | Y | | Colombia | | G | | (Ormeño-Orrillo et al., 2012) | |

^a^ According to Figure 1

Althabegoiti, M.J., Lozano, L., Torres-Tejerizo, G., Ormeño-Orrillo, E., Rogel, M.A., González, V., and Martínez-Romero, E. (2012). Genome sequence of *Rhizobium grahamii* CCGE502, a broad-host-range symbiont with low nodulation competitiveness in *Phaseolus vulgaris*. *Journal of Bacteriology* 194**,** 6651-6652.

Aserse, A.A., Woyke, T., Kyrpides, N.C., Whitman, W.B., and Lindström, K. (2017). Draft genome sequence of type strain HBR26(T) and description of *Rhizobium aethiopicum* sp. nov. *Standards in Genomic Sciences* 12**,** 14.

De Meyer, S.E., Tian, R., Seshadri, R., Ivanova, N., Pati, A., Markowitz, V., Woyke, T., Yates, R., Howieson, J., Kyrpides, N., and Reeve, W. (2015a). High-quality permanent draft genome sequence of the *Lebeckia* - nodulating *Burkholderia dilworthii* strain WSM3556T. *Standards in Genomic Sciences* 10**,** 64.

De Meyer, S.E., Tian, R., Seshadri, R., Reddy, T.B.K., Markowitz, V., Ivanova, N., Pati, A., Woyke, T., Kyrpides, N., Yates, R., Howieson, J., and Reeve, W. (2015b). High-quality permanent draft genome sequence of the *Lebeckia ambigua*-nodulating *Burkholderia* sp. strain WSM4176. *Standards in Genomic Sciences* 10**,** 79.

Galardini, M., Bazzicalupo, M., Biondi, E., Brambilla, E., Brilli, M., Bruce, D., Chain, P., Chen, A., Daligault, H., Davenport, K.W., Deshpande, S., Detter, J.C., Goodwin, L.A., Han, C., Han, J., Huntemann, M., Ivanova, N., Klenk, H.-P., Kyrpides, N.C., Markowitz, V., Mavrommatis, K., Mocali, S., Nolan, M., Pagani, I., Pati, A., Pini, F., Pitluck, S., Spini, G., Szeto, E., Teshima, H., Woyke, T., and Mengoni, A. (2013a). Permanent draft genome sequences of the symbiotic nitrogen fixing *Ensifer meliloti* strains BO21CC and AK58. *Standards in Genomic Sciences* 9**,** 325-333.

Galardini, M., Mengoni, A., Brilli, M., Pini, F., Fioravanti, A., Lucas, S., Lapidus, A., Cheng, J.-F., Goodwin, L., Pitluck, S., Land, M., Hauser, L., Woyke, T., Mikhailova, N., Ivanova, N., Daligault, H., Bruce, D., Detter, C., Tapia, R., Han, C., Teshima, H., Mocali, S., Bazzicalupo, M., and Biondi, E.G. (2011). Exploring the symbiotic pangenome of the nitrogen-fixing bacterium *Sinorhizobium meliloti*. *BMC Genomics* 12**,** 235-235.

Galardini, M., Pini, F., Bazzicalupo, M., Biondi, E.G., and Mengoni, A. (2013b). Replicon-dependent bacterial genome evolution: the case of *Sinorhizobium meliloti*. *Genome Biology and Evolution* 5**,** 542-558.

Galibert, F., Finan, T.M., Long, S.R., Pühler, A., Abola, P., Ampe, F., Barloy-Hubler, F., Barnett, M.J., Becker, A., Boistard, P., Bothe, G., Boutry, M., Bowser, L., Buhrmester, J., Cadieu, E., Capela, D., Chain, P., Cowie, A., Davis, R.W., Dréano, S., Federspiel, N.A., Fisher, R.F., Gloux, S., Godrie, T., Goffeau, A., Golding, B., Gouzy, J., Gurjal, M., Hernandez-Lucas, I., Hong, A., Huizar, L., Hyman, R.W., Jones, T., Kahn, D., Kahn, M.L., Kalman, S., Keating, D.H., Kiss, E., Komp, C., Lelaure, V., Masuy, D., Palm, C., Peck, M.C., Pohl, T.M., Portetelle, D., Purnelle, B., Ramsperger, U., Surzycki, R., Thébault, P., Vandenbol, M., Vorhölter, F.-J., Weidner, S., Wells, D.H., Wong, K., Yeh, K.-C., and Batut, J. (2001). The composite genome of the legume symbiont *Sinorhizobium meliloti*. *Science* 293**,** 668-672.

González, V., Santamaría, R.I., Bustos, P., Hernández-González, I., Medrano-Soto, A., Moreno-Hagelsieb, G., Janga, S.C., Ramírez, M.A., Jiménez-Jacinto, V., Collado-Vides, J., and Dávila, G. (2006). The partitioned *Rhizobium etli* genome: Genetic and metabolic redundancy in seven interacting replicons. *Proceedings of the National Academy of Sciences of the United States of America* 103**,** 3834-3839.

Hou, B.C., Wang, E.T., Li, Y., Jia, R.Z., Chen, W.F., Gao, Y., Dong, R.J., and Chen, W.X. (2009). *Rhizobium tibeticum* sp. nov., a symbiotic bacterium isolated from *Trigonella archiducis*-*nicolai* (Širj.) Vassilcz. *International Journal of Systematic and Evolutionary Microbiology* 59**,** 3051-3057.

Klonowska, A., López-López, A., Moulin, L., Ardley, J., Gollagher, M., Marinova, D., Tian, R., Huntemann, M., Reddy, T.B.K., Varghese, N., Woyke, T., Markowitz, V., Ivanova, N., Seshadri, R., Baeshen, M.N., Baeshen, N.A., Kyrpides, N., and Reeve, W. (2017). High-quality draft genome sequence of *Rhizobium mesoamericanum* strain STM6155, a *Mimosa pudica* microsymbiont from New Caledonia. *Standards in Genomic Sciences* 12**,** 7.

Li, Z., Ma, Z., Hao, X., and Wei, G. (2012). Draft genome sequence of *Sinorhizobium meliloti* CCNWSX0020, a nitrogen-fixing symbiont with copper tolerance capability isolated from lead-zinc mine tailings. *Journal of Bacteriology* 194**,** 1267-1268.

López-Guerrero, M.G., Ormeño-Orrillo, E., Velázquez, E., Rogel, M.A., Acosta, J.L., Gónzalez, V., Martínez, J., and Martínez-Romero, E. (2012). *Rhizobium etli* taxonomy revised with novel genomic data and analyses. *Systematic and Applied Microbiology* 35**,** 353-358.

Martínez-Abarca, F., Martínez-Rodríguez, L., López-Contreras, J.A., Jiménez-Zurdo, J.I., and Toro, N. (2013). Complete genome sequence of the alfalfa symbiont *Sinorhizobium/Ensifer meliloti* strain GR4. *Genome Announcements* 1**,** e00174-00112.

Moulin, L., Mornico, D., Melkonian, R., and Klonowska, A. (2013). Draft genome sequence of *Rhizobium mesoamericanum* STM3625, a nitrogen-fixing symbiont of *Mimosa pudica* isolated in French Guiana (South America). *Genome Announcements* 1**,** e00066-00012.

Ormeño-Orrillo, E., Menna, P., Almeida, L.G.P., Ollero, F.J., Nicolás, M.F., Pains Rodrigues, E., Shigueyoshi Nakatani, A., Silva Batista, J.S., Oliveira Chueire, L.M., Souza, R.C., Ribeiro Vasconcelos, A.T., Megías, M., Hungria, M., and Martínez-Romero, E. (2012). Genomic basis of broad host range and environmental adaptability of *Rhizobium tropici* CIAT 899 and *Rhizobium* sp. PRF 81 which are used in inoculants for common bean (*Phaseolus vulgaris* L.). *BMC Genomics* 13**,** 1-26.

Osman, W.a.M., Van Berkum, P., León-Barrios, M., Velázquez, E., Elia, P., Tian, R., Ardley, J., Gollagher, M., Seshadri, R., and Reddy, T. (2017). High-quality draft genome sequence of Ensifer meliloti Mlalz-1, a microsymbiont of Medicago laciniata (L.) miller collected in Lanzarote, Canary Islands, Spain. *Standards in genomic sciences* 12**,** 1-12.

Reeve, W., Ballard, R., Drew, E., Tian, R., Bräu, L., Goodwin, L., Huntemann, M., Han, J., Tatiparthi, R., Chen, A., Mavrommatis, K., Markowitz, V., Palaniappan, K., Ivanova, N., Pati, A., Woyke, T., and Kyrpides, N. (2014). Genome sequence of the *Medicago*-nodulating *Ensifer meliloti* commercial inoculant strain RRI128. *Standards in Genomic Sciences* 9**,** 602-613.

Reeve, W.G., Ardley, J., Tian, R., Eshragi, L., Yoon, J.W., Ngamwisetkun, P., Seshadri, R., Ivanova, N.N., and Kyrpides, N.C. (2015). A genomic encyclopedia of the root nodule bacteria: Assessing genetic diversity through a systematic biogeographic survey. *Standards in Genomic Sciences* 10:14.

Sallet, E., Roux, B., Sauviac, L., Jardinaud, M.-F.O., Carrère, S., Faraut, T., De Carvalho-Niebel, F., Gouzy, J., Gamas, P., Capela, D., Bruand, C., and Schiex, T. (2013). Next-generation annotation of prokaryotic genomes with EuGene-P: application to *Sinorhizobium meliloti* 2011. *DNA Research* 20**,** 339-354.

Schneiker-Bekel, S., Wibberg, D., Bekel, T., Blom, J., Linke, B., Neuweger, H., Stiens, M., Vorhölter, F.-J., Weidner, S., Goesmann, A., Pühler, A., and Schlüter, A. (2011). The complete genome sequence of the dominant *Sinorhizobium meliloti* field isolate SM11 extends the *S. meliloti* pan-genome. *Journal of Biotechnology* 155**,** 20-33.

Terpolilli, J., Hill, Y., Tian, R., Howieson, J., Bräu, L., Goodwin, L., Han, J., Liolios, K., Huntemann, M., Pati, A., Woyke, T., Mavromatis, K., Markowitz, V., Ivanova, N., Kyrpides, N., and Reeve, W. (2013). Genome sequence of *Ensifer meliloti* strain WSM1022; a highly effective microsymbiont of the model legume *Medicago truncatula* A17. *Standards in Genomic Sciences* 9**,** 315-324.

Torres Tejerizo, G., Lozano, L., González, V., Bustos, P., Romero, D., and Brom, S. (2012). Draft genome sequence of the bean-nodulating *Sinorhizobium fredii* strain GR64. *Journal of Bacteriology* 194**,** 6978.

Torres Tejerizo, G., Wibberg, D., Winkler, A., Ormeño-Orrillo, E., Martínez-Romero, E., Niehaus, K., Pühler, A., Kalinowski, J., Lagares, A., Schlüter, A., and Pistorio, M. (2017). Genome sequence of the symbiotic type strain *Rhizobium tibeticum* CCBAU85039(T). *Genome Announcements* 5**,** e01513-01516.

Valverde, A., Igual, J.M., Peix, A., Cervantes, E., and Velázquez, E. (2006). *Rhizobium lusitanum* sp. nov. a bacterium that nodulates *Phaseolus vulgaris*. *International Journal of Systematic and Evolutionary Microbiology* 56**,** 2631-2637.

Weidner, S., Baumgarth, B., Göttfert, M., Jaenicke, S., Pühler, A., Schneiker-Bekel, S., Serrania, J., Szczepanowski, R., and Becker, A. (2013). Genome sequence of *Sinorhizobium meliloti* Rm41. *Genome Announcements* 1**,** e00013-00012.

Whitman, W.B., Woyke, T., Klenk, H.-P., Zhou, Y., Lilburn, T.G., Beck, B.J., De Vos, P., Vandamme, P., Eisen, J.A., and Garrity, G. (2015). Genomic encyclopedia of bacterial and archaeal type strains, phase III: the genomes of soil and plant-associated and newly described type strains. *Standards in genomic sciences* 10**,** 1-6.

Wibberg, D., Tejerizo, G.T., Del Papa, M.F., Martini, C., Puhler, A., Lagares, A., Schluter, A., and Pistorio, M. (2014). Genome sequence of the acid-tolerant strain *Rhizobium* sp LPU83. *Journal of Biotechnology* 176**,** 40-41.
